# Transient “rest” restores functionality in exhausted CAR-T cells via epigenetic remodeling

**DOI:** 10.1126/science.aba1786

**Published:** 2021-04-02

**Authors:** Evan W. Weber, Kevin R. Parker, Elena Sotillo, Rachel C. Lynn, Hima Anbunathan, John Lattin, Zinaida Good, Julia A. Belk, Bence Daniel, Dorota Klysz, Meena Malipatlolla, Peng Xu, Malek Bashti, Sabine Heitzeneder, Louai Labanieh, Panayiotis Vandris, Robbie G. Majzner, Yanyan Qi, Katalin Sandor, Ling-Chun Chen, Snehit Prabhu, Andrew J. Gentles, Thomas J. Wandless, Ansuman T. Satpathy, Howard Y. Chang, Crystal L. Mackall

**Affiliations:** 1 Center for Cancer Cell Therapy, Stanford Cancer Institute, Stanford University School of Medicine, Stanford, CA, 94305; 2 Department of Personal Dynamic Regulomes, Stanford University School of Medicine, Stanford, CA, 94305; 3 Parker Institute for Cancer Immunotherapy, San Francisco, CA, 941209; 4 Department of Computer Science, Stanford University, Stanford, CA, 94305; 5 Department of Pathology, Stanford University, Stanford, CA, 94305; 6 Department of Pediatrics, Stanford University School of Medicine, Stanford, CA, 94305; 7 Department and Chemical and Systems Biology, Stanford University, CA, 94305; 8 Department of Biomedical Informatics Research, Stanford University School of Medicine, Stanford, CA, 94305; 9 Howard Hughes Medical Institute, Stanford University, Stanford, CA, 94305; 10 Department of Medicine, Stanford University School of Medicine, Stanford, CA, 94305

## Abstract

T cell exhaustion limits immune responses against cancer and is a major cause of resistance to chimeric antigen receptor (CAR) T cell therapeutics. Using xenograft models and an in vitro model wherein tonic CAR signaling induces hallmark features of exhaustion, we tested the impact of transient cessation of receptor signaling, or “rest”, on the development and maintenance of exhaustion. Induction of rest via enforced CAR protein downregulation using a drug-regulatable system or treatment with the multi-kinase inhibitor dasatinib resulted in acquisition of a memory-like phenotype, wholescale transcriptional and epigenetic reprogramming, and restored anti-tumor functionality in exhausted CAR-T cells. This work demonstrates that rest can enhance CAR-T cell efficacy by preventing or reversing exhaustion and challenges the notion that exhaustion is an epigenetically fixed state.

## INTRODUCTION

Chimeric antigen receptors (CARs) combine a tumor antigen-recognition domain with intracellular signaling domains, enabling recognition and killing of tumor cells in a major histocompatibility complex (MHC)-independent manner (1). CAR-T cells mediate high response rates in relapsed/refractory high-grade B cell malignancies, but less than 50% of patients experience long-term disease control and CAR-T cells have not demonstrated reproducible efficacy against solid tumors (2, 3). T cell exhaustion has been implicated as an important factor limiting the efficacy of CAR-T cells against cancer (4-6) and can be driven by excessive CAR signaling as a result of high antigen burden or tonic signaling induced by antigen-independent clustering of the CAR receptor (7, 8). We hypothesized that transient cessation of CAR signaling would enable exhausted CAR-T cells to regain functionality and form a memory pool, similar to the effects observed following antigen clearance of acute infections (9).

Using a drug-regulatable platform (10, 11) wherein a tonically signaling CAR was modified with a C-terminal destabilizing domain (DD) to enable drug-dependent control of CAR protein levels, we observed that transient inhibition of CAR surface expression (“rest”), and thereby tonic CAR signaling, prevented cells from developing phenotypic hallmarks of exhaustion, instead redirecting them to a memory-like fate. Furthermore, transient rest, but not PD-1 blockade, restored anti-tumor functionality in a cell population that had already acquired functional, transcriptional and epigenetic features of exhaustion, and was associated with global transcriptional and epigenetic reprogramming. We observed similar phenotypic and functional reinvigoration following transient exposure to dasatinib, a tyrosine kinase inhibitor that reversibly inhibits TCR and CAR signaling (12-15). Collectively, these results challenge the concept that exhaustion is an epigenetically fixed state (16, 17) and reveal that transient cessation of CAR signaling may provide a strategy for augmenting function of exhausted human CAR-T cell populations.

## RESULTS

### CARs modified with a destabilizing domain exhibits drug-dependent, tunable control of expression and function in vitro and in vivo.

**Figure 1:**
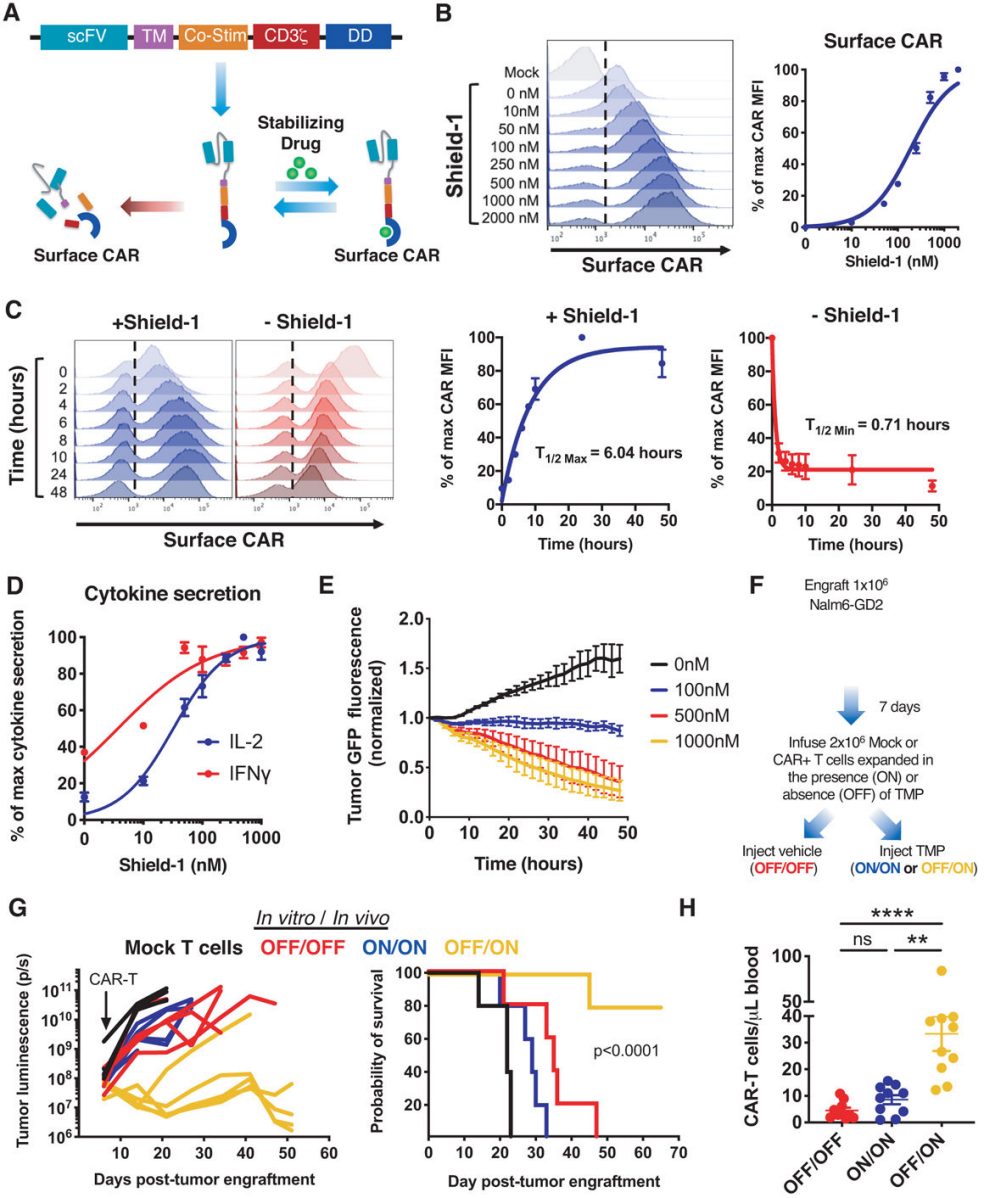
A GD2-targeting CAR modified with a destabilizing domain (DD) exhibits drug-dependent control of expression, function, and tonic CAR signaling. **A)** Schematic depicting drug-dependent control of DD CAR fusion protein. **B)** Flow cytometric analysis of GD2.28ζ.FKBP CAR surface expression at increasing concentrations of shield-1. **C)** Flow cytometric analysis of ON/OFF kinetics of GD2.28ζ.FKBP CAR surface expression at indicated time points after addition or removal shield-1. **D)** IL-2 and IFNγ secretion of GD2.28ζ.FKBP CAR-T cells pretreated with indicated concentrations of shield-1 16 hours prior to co-culture with Nalm6-GD2 leukemia. **E)** Cytotoxicity of GD2.28ζ.FKBP CAR-T cells treated as in (D) against Nalm6-GD2-GFP leukemia (1:2 E:T, normalized to t=0). Error bars represent mean ± SD of triplicate wells. Representative donor from 3 donors. **F-H)** 2x10^6^ GD2.28ζ.ecDHFR CAR-T cells expanded in the presence or absence of trimethoprim (TMP) for 15 days in vitro **(F)** were infused IV in NSG mice 7 days post-engraftment of 1x10^6^ Nalm6-GD2 leukemia cells. Mice were dosed 6 days per week with vehicle (water, OFF/OFF) or 200mg/kg TMP (ON/ON and OFF/ON). **(G)** Quantification of tumor growth by bioluminescent imaging (right) and survival (left) (p<0.0001 log-rank Mantel-Cox test). Representative experiment from 3 independent experiments (n=5 mice/group). **(H)** Detection of CAR-T cells in peripheral blood sampled on day 28 post-engraftment by flow cytometry after anti-human CD45 staining (n=10 mice/group from 2 independent experiments). B-C show histograms from one representative donor (n=3 donors). Curves in B-D show mean ± SEM from 3 donors. Statistics: **G** log-rank Mantel-Cox test, **H** Kruskal-Wallis and Dunn’s multiple comparisons test. **, p<0.01; ****p<0.0001; ns, p>0.05

We previously demonstrated that some CARs undergo antigen-independent, tonic CAR signaling due to spontaneous receptor clustering, which promotes hallmark features of exhaustion in human T cells (7, 8). To test whether prevention of tonic signaling preserves CAR-T cell functionality, we incorporated an FK506 binding protein 12 (FKBP) destabilizing domain (DD) (11) into a tonically signaling GD2-targeting CAR (GD2.28ζ.FKBP), similar to a recently published study (18). The DD induced rapid degradation of the CAR at baseline (Fig. 1, A and B), whereas shield-1, which stabilizes the FKBP DD, prevented CAR degradation thereby inducing GD2.28ζ.FKBP CAR expression on the cell surface in a dose- and time-dependent manner (Fig. 1, B and C). Removal of shield-1 led to a rapid decrease in CAR surface protein, with a degradation half-life of approximately 1 hour (Fig. 1C). To determine whether the dynamic range of CAR expression induced by the DD modulated biologic reactivity and to assess the relationship between CAR expression level and function, we co-cultured GD2.28ζ.FKBP CAR-T cells with Nalm6 leukemia engineered to express GD2, GFP, and luciferase (Nalm6-GD2) with increasing concentrations of shield-1. We observed drug-dependent, tunable control of tumor-induced cytokine secretion (Fig. 1D) and cytotoxicity (Fig. 1E), demonstrating that modulation of CAR expression levels tuned CAR-T cell function.

To interrogate DD-CAR functionality in vivo, we engineered a GD2.28ζ CAR incorporating an *E. coli*-derived dihydrofolate reductase DD (GD2.28ζ.ecDHFR) (19), which is regulated by the FDA-approved antibiotic, trimethoprim (TMP), and demonstrated a dynamic range comparable to that of the GD2.28ζ.FKBP CAR (fig. S1A). In NOD/SCID/IL2Rγ^−/−^ (NSG) mice engrafted with Nalm6-GD2 leukemia, intraperitoneal TMP administration upregulated surface CAR and CD69 in CAR-T cells isolated from blood and spleen compared to controls (fig. S1B). Together, these data demonstrate drug-dependent control of DD-CAR expression and activity in vitro and in vivo.

### Cessation of tonic CAR signaling augments CAR-T cell functionality and redirects CAR-T cell fate away from exhaustion and towards a memory-like state.

We next sought to determine whether control of DD-CAR expression prevented exhaustion induced by tonic CAR signaling. GD2.28ζ.FKBP CAR-T cells cultured with shield-1 (ON) exhibited antigen-independent phosphorylation of CAR CD3ζ (CD3ζ) (fig. S1C) and elevated expression of inhibitory receptors PD-1, TIM-3, and LAG-3 (fig. S1D), compared to those cultured without shield-1 (OFF). GD2.28ζ.FKBP or GD2.28ζ.ecDHFR T cells expanded in the OFF state, but provided drug just prior to antigen challenge (OFF/ON) exhibited superior cytotoxicity (fig. S1E), tumor-induced cytokine secretion (fig. S1F), and enhanced efficacy and cell expansion in vivo (Fig. 1, F and G). Collectively, these observations confirm that tonic CAR signaling induces T cell dysfunction and demonstrate that prevention of tonic signaling ex vivo augments CAR-T cell efficacy.

**Figure 2:**
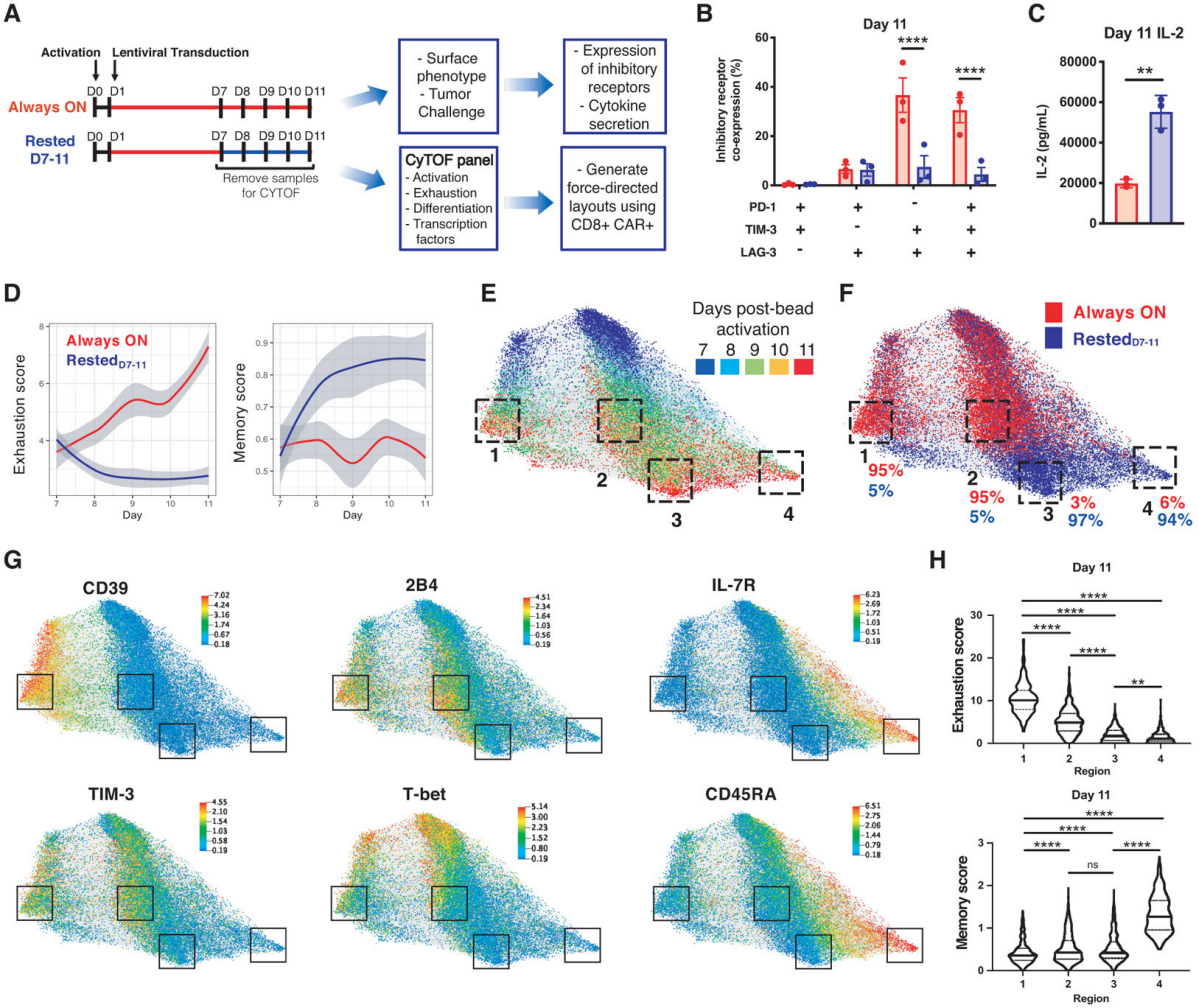
Cessation of tonic CAR signaling redirects CAR-T cell fate. **A)** Experimental design: HA.28ζ.FKBP CAR-T cells were cultured in the presence of shield-1 from D1-11 (Always ON) or treatment was discontinued on D7 (Rested_D7-11_) **B)** Co-expression of inhibitory receptors by flow cytometry on D11. Error bars represent mean ± SEM of 3 donors. **C)** IL-2 secretion in response to Nalm6-GD2 leukemia on D11. Error bars represent mean ± SD of triplicate wells from one representative donor (n=5 donors). **D-H)** Force-directed layout (FDL) constructed from 20,000 CAR+/CD8+ events analyzed by mass cytometry. Cells repel based on expression of 20 surface and 2 intracellular markers (Figure S2B) and grey edges connect cells from adjacent days (representative donor from n=3 donors). (D) Local polynomial regression fitting exhaustion (left panel) and memory (right panel) scores in 2,000 sampled cells from Always ON and Rested_D7-11_ conditions over time. Shaded regions indicate 95% confidence intervals. Refer to Methods for details. (E) FDL colored by timepoint. D11 cells are concentrated in phenotypically distinct regions 1-4 (Figure S2C). (F) FDL colored by treatment. Percent of Always ON and Rested_D7-11_ cells is shown for each D11 region. (G) FDL colored by expression of indicated proteins. (H) Violin plots of D11 cell exhaustion and memory scores contained within regions 1-4 show quartiles with a band at the mean. Statistics: **B t**wo-way ANOVA with Dunnett’s multiple comparisons test, **C** unpaired two-tailed student’s t test, **H** or Kruskal-Wallis with Dunn’s multiple comparisons test. **, p<0.01; ****, p<0.0001; ns, p>0.05

We next regulated CAR expression in HA.28ζ-CAR-T cells (fig. S2A), which manifest extremely robust tonic signaling and acquire functional, transcriptomic and epigenetic hallmarks of exhaustion by D11 (*8*). When compared to HA.28ζ.FKBP CAR-T cells continuously cultured with shield-1 (Always ON), cells from which shield-1 was removed on D7 (Rested_D7-11_) demonstrated decreased inhibitory receptor expression and superior functionality upon tumor challenge on D11 (Fig. 2, A through C). Mass cytometric analysis of 27 proteins associated with T cell exhaustion, activation, or memory (fig. S2B, Table S1) demonstrated that CD8+ Always ON cells manifested time-dependent increases in exhaustion scores (normalized mean expression of PD-1, TIM-3, LAG-3, CTLA-4, BTLA, 2B4, and CD39), whereas Rested_D7-11_ cells demonstrated time-dependent increases in memory scores (normalized mean expression of CD45RA, IL-7R, CD27, CD197) (Fig. 2D).

Force-directed layouts (FDLs) (20), which map phenotypically similar cells closely together and dissimilar cells farther apart, illustrated trajectories towards exhaustion or memory-like cell fates. CD8+ Always ON and Rested_D7-11_ CAR-T cells displayed substantial evolution during D7-11 and disparate phenotypes by D11 were spatially distributed between 4 distinct regions on the FDL (Fig. 2E and fig. S2C). The majority of D11 Always ON cells resided in Regions 1 or 2, while the majority of Rested_D7-11_ cells resided in Regions 3 or 4 (Fig. 2F). FDLs also revealed heterogeneity within the exhausted and memory subsets, with Region 1 demonstrating a higher exhaustion score and increased CD39 compared to Region 2 (fig. S2D), and Region 4 demonstrating a higher memory score and increased expression of IL7RA and CD45RA compared to Region 3 (Fig. 2, G and H, and fig. S2E). Rested_D7-11_ cells exhibited reduced T-bet and Blimp-1 expression levels and co-expression frequency (fig. S2F), consistent with transcriptional reprogramming (21, 22).

Redirection of cell fate induced by rest from D7-11 was most likely due to population-wide changes rather than outgrowth of a rare subpopulation since we detected only modest differences in fold change in cell expansion, Ki-67 expression, and cleaved PARP (cPARP) between Always ON and Rested_D7-11_ cells (fig. S2, G and H). Moreover, TCF1 expression, which is associated with a progenitor exhausted cell population that retains anti-tumor functionality and is responsive to checkpoint blockade (23-26), was similar in Always ON and Rested_D7-11_ conditions, representing approximately 10% of total CD8+ CAR-T cells on D11 (fig. S2I). RNA-sequencing at D11 demonstrated that both Always ON and Rested_D7-11_ cells contained ~1,000 unique TCR clonotypes with similar clonotypic diversity (fig. S2, J and K). Similar phenotypic changes were observed in Rested_D7-11_ CD4+ CAR-T cells (fig. S3). Collectively, these observations indicate that transient cessation of CAR signaling prior to exhaustion onset alters the differentiation trajectory of a large fraction of CAR-T cell populations rather than inducing outgrowth of a minor subset of highly proliferative, apoptosis-resistant, or TCF1+ progenitor exhausted Rested_D7-11_ cells.

### Transient rest reverses phenotypic and transcriptomic hallmarks of exhaustion

**Figure 3:**
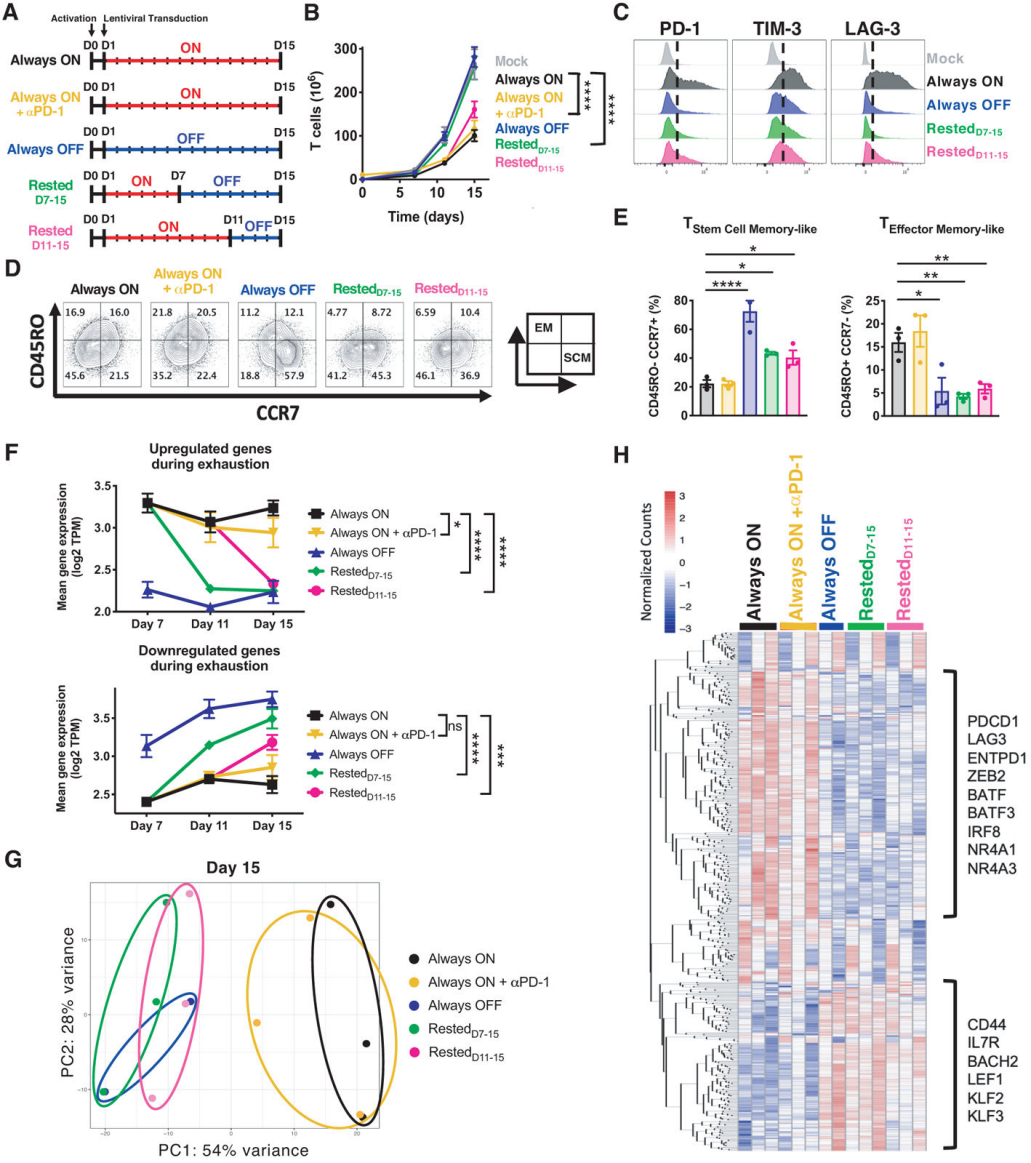
Transient rest reverses phenotypic and transcriptomic hallmarks of exhaustion. **A)** Experimental design: Activated human T cells were transduced with HA.28ζ.FKBP CAR lentivirus on D1. CAR-T cells were cultured in the presence (red) or absence (blue) of stabilizing drug for the indicated periods of time. Cells were collected on D7, D11, and D15 for FACS and bulk RNA sequencing. **B)** Cell growth curves (n=6-9 donors). **C-E)** Flow cytometric analysis of the expression of (C) inhibitory receptors and (D) effector or (E) stem cell memory markers on CD8+ CAR+ T cells. Histograms and contour plots of representative donor are shown. Bar graphs show mean ± SEM of 3 independent donors. Inset in C illustrates effector-memory-like (EM, top-left) and stem cell memory-like (SCM, bottom-right) phenotype quadrants. **F-H)** Bulk RNA sequencing analyses of mixed CD4+/CD8+ HA.28ζ.FKBP CAR-T cells. (F) Kinetics of the mean expression of genes upregulated (top) or downregulated (bottom) during exhaustion at each timepoint for every experimental group (Fig. S4G). Error bars represent the mean ± SEM of 2-3 independent donors. Two-way ANOVA demonstrates significant differences in mean exhaustion signature gene expression between Always ON and rested conditions on D15. (G) Unbiased principal component analysis of D15 cells. (H) Heatmap and hierarchical clustering of the top 500 genes driving principal component 1 (PC1), which identified clusters of exhaustion- and memory-associated genes that change in rested cells. Statistics: **B** paired two-tailed student’s t test, **E and F** one- or two-way ANOVA with Dunnett’s multiple comparisons test. *, p<0.05; **, p<0.01; ***, p<0.001; ****, p<0.0001; ns, p>0.05

Since cessation of tonic signaling altered CAR-T cell fate during the transition to exhaustion, we hypothesized that rest could also reprogram T cell populations on which exhaustion is already imprinted. We compared D15 Always ON HA.28ζ.FKBP CAR-T cells to cells rested from D11-15 (Rested_D11-15_, Fig. 3A). PD-1 and PD-L1 are both expressed on activated T cells (fig. S2E), and in some experiments we cultured Always ON T cells with αPD-1 from D7-15 to compare the effects of checkpoint blockade with that of transient rest. As expected, D15 Always ON cells exhibited robust tonic CAR signaling, poor expansion, elevated immune checkpoint receptors (PD-1, TIM-3 and LAG-3), and an effector-like phenotype (Fig. 3, B through E, and fig. S4, A through F) compared to Always OFF cells. In contrast, CD8+ Rested_D11-15_ cells displayed diminished tonic signaling, reduction in inhibitory receptor expression, increased stem cell memory-like cells (CD45RO−, CCR7+), and enhanced proliferation compared to Always ON and Always ON + αPD-1 T cells. Sequential RNA sequencing demonstrated that transcripts associated with exhaustion underwent rapid and complete reversal to baseline Always OFF levels in rested cells by D11 or D15, whereas αPD-1 in Always ON T cell cultures induced only a small but detectable effect on gene expression (Fig. 3F and fig. S4G).

Unbiased principal component analysis (PCA) at each timepoint displayed overlap between Always OFF and rested conditions, which separated from Always ON and Always ON + αPD-1 cells along PC1 (Fig. 3G and fig. S4H), illustrating the high degree of transcriptomic reprogramming induced by rest. Hierarchical clustering of the top 500 genes driving PC1 variance identified exhaustion- (*PDCD1, ENTPD1, BATF, NR4A1*) and memory-associated genes (*IL7R, LEF1, KLF2, BACH2)*(Fig. 3H). D15 analyses confirmed transcriptomic reversal, since Rested_D11-15_ T cells significantly upregulated memory/quiescence-associated genes (*SELL, LEF1, FOXO3*) and downregulated canonical exhaustion-associated genes (*CTLA-4, IRF4, TOX2, NR4A3*) compared to Always ON T cells (p<0.05, fig. S4, I and J). Importantly, both exhausted and rested populations on D15 contained ~1,000-3,000 unique TCR clonotypes and exhibited similar clonotypic diversity and rates of apoptosis (fig. S4, K through M). These data are consistent with rest-induced changes occurring broadly within the population under study, rather than preferential expansion of a small subset of cells, which would result in reduced clonotypic diversity.

### Transient rest reinvigorates exhausted CAR-T cells and improves therapeutic efficacy

**Figure 4:**
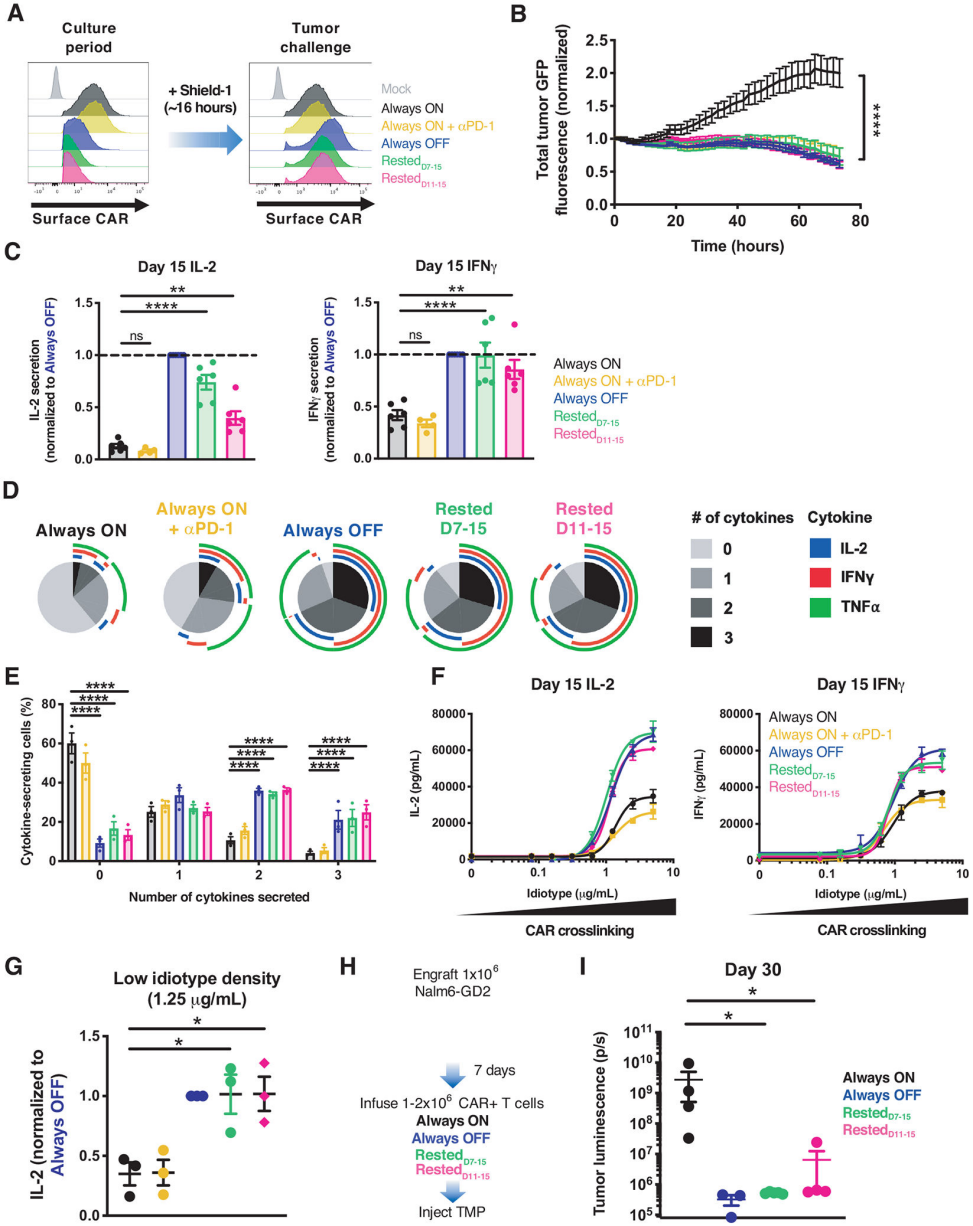
Transient rest reinvigorates exhausted CAR-T cells and improves anti-tumor function. **A)** Flow cytometric analysis of HA.28ζ.FKBP CAR expression before and after treatment with shield-1. **B)** Cytotoxic activity of D15 HA.28ζ.FKBP CAR-T cells from each treatment group against 143B-GL osteosarcoma (1:8 E:T, normalized to t=0). **C)** IL-2 (left) and IFNγ (right) secretion of D15 HA.28ζ.FKBP CAR-T cells co-cultured with 143B-GL osteosarcoma cells. Data were normalized to Always OFF values. **D-E)** Flow cytometry analyses after intracellular cytokine staining of CD8+ CAR+ T cells activated with 143B-GL. (D) shows SPICE analysis from 1 representative donor and (E) shows polyfunctionality. **F-G)** IL-2 and IFNγ secretion in response to crosslinking with increasing concentrations of immobilized 1A7 anti-CAR idiotype antibody for 24 hours. (F) shows non-linear dose-response curves and (G) shows IL-2 secretion in response to low density (1.25 μg/mL) idiotype where secretion levels were normalized to Always OFF. **H-I)** 1-2x10^6^ HA.28ζ.ecDHFR CAR-T cells expanded for 15 days in vitro (as depicted in Fig. 4A) (H) were infused IV 7 days post-engraftment of 1x10^6^ Nalm6-GD2 leukemia cells. Mice were dosed 200 mg/kg TMP 6 days/week. **(I)** Bioluminescent imaging of tumor growth 30 days post-engraftment. Error bars represent mean ± SEM of 3-5 mice from 1 representative experiment (n=3 independent experiments). In B and F error bars represent mean ± SD of 3 triplicate wells from one representative donor (n=3 donors). Error bars represent mean ± SEM of 4-6 individual donors in C and of 3 donors in E and G. Statistics: **B, C, E, G** one or two-way ANOVA with Dunnett’s multiple comparisons test, **I** Mann-Whitney test. *, p<0.05; **, p<0.01; ****, p<0.0001; ns, p>0.05

We next tested whether the phenotypic and transcriptomic reprogramming of exhausted CAR-T cells induced by rest would confer enhanced anti-tumor functionality. Prior to antigen challenge, we treated CAR-T cells with shield-1 for 16 hours to normalize CAR surface expression (Fig. 4A). On D15, Rested_7-15_ and Rested_11-15_ T cells demonstrated marked enhancement in cytotoxicity and cytokine secretion compared to exhausted Always ON cells (Fig. 4, B and C, and fig. S5A). Single cell analyses revealed that approximately 60% of Always OFF and rested CAR-T cells were polyfunctional and capable of secreting at least two cytokines, whereas less than 20% of Always ON T cells exhibited these features (Fig. 4, D and E, and fig. S5, B and C), demonstrating functional reinvigoration in a high fraction of rested T cells. In contrast to rest, PD-1 blockade enhanced cytotoxicity but did not significantly augment IL-2 or IFNγ secretion, the frequency of cytokine-secreting cells, or sensitivity to low antigen (Fig. 4, B through G, and fig. S5, B and C), indicating that rest-associated functional reinvigoration is mechanistically distinct from that of checkpoint blockade. In vivo studies corroborated these findings (Fig. 4H and fig. S5, D and E), since exhausted Always ON T cells failed to control tumor growth, whereas Rested_D7-15_, and Rested_D11-15_ T cells cured or maintained the tumor at levels comparable to Always OFF (Fig. 4I, and fig. S5F). These data demonstrate that transient cessation of CAR signaling reverses the exhaustion phenotype and rescues CAR-T cell functionality.

### Rest induces wholescale remodeling of the exhaustion-associated epigenome

**Figure 5:**
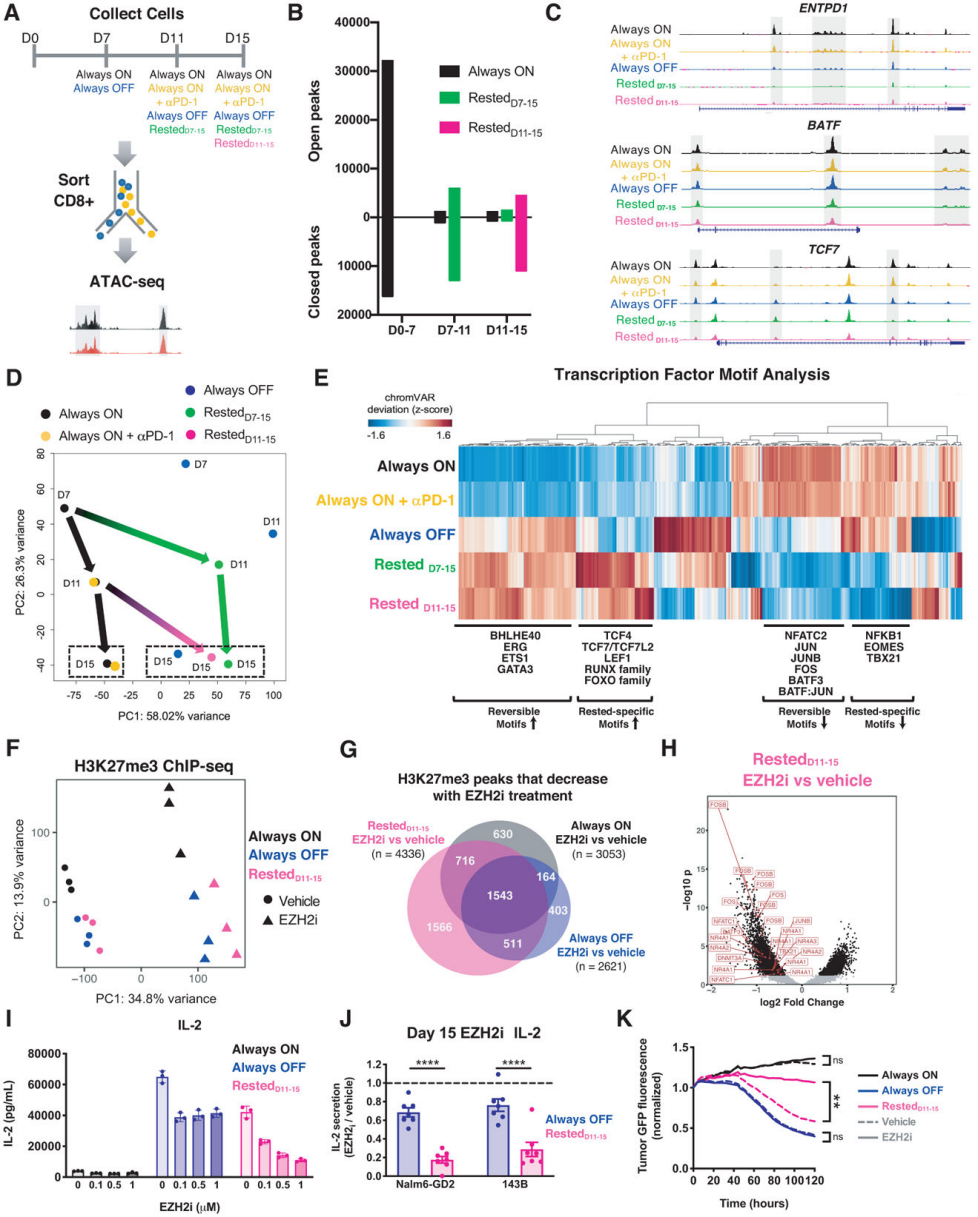
Rested CAR-T cells exhibit wholescale remodeling of the exhaustion-associated epigenome. **A)** Experimental design and sample processing for ATAC-seq analyses. **B)** Peak accessibility changes between timepoints calculated based on p adjusted<0.05. Merged data from 2-3 donors is shown. **C)** Accessibility profiles in the *ENTPD1, BATF*, and *TCF7* loci on D15. Representative donor (n=2-3 donors). **D)** Unbiased PCA of chromatin accessibility assessed across all timepoints. Green and magenta arrows indicate global epigenetic remodeling upon rest. Dashed boxes indicate D15 samples. Each dot represents merged data from 2-3 donors. **E)** Hierarchical clustering of differentially accessible TF motifs in D15 samples. The heatmap was generated using merged data from 2-3 donors. **F-H)** H3K27me3 chromatin immunoprecipitation sequencing on D15 CD8+ CAR-T cells (n=3 donors) from Always ON, Always OFF, and Rested_D11-15_ CAR-T cells treated with 0.1-1 μM (F) or 1 μM (G-K) tazemetostat (EZH2i) or vehicle from D11-15. **F)** PCA across all treatment groups. **G)** Venn diagrams showing differentially decreased H3K27me3 peaks in EZH2i-treated samples compared to vehicle. Numbers indicate unique peaks. **H)** Volcano plot of differentially decreased H3K27me3 peaks (filtered for p(adjusted)<0.05) in EZH2_i_-treated Rested_D11-15_ cells compared to controls. **I)** IL-2 secretion in CAR-T cells treated with increasing concentrations of tazemetostat in response to Nalm6-GD2 (mean ± SD of triplicate wells from 1 representative donor of n=4 donors). **J)** IL-2 secretion in response to Nalm6-GD2 or 143B-GL normalized to vehicle controls (mean ± SEM of n=7 individual donors). **K)** Cytotoxic activity against 143B-GL tumor (1:4 E:T, normalized to t=0). Mean of triplicate wells from 1 representative donor is shown (n=3 donors). Statistics: two-way ANOVA with Bonferroni’s **(J)** or Dunnett’s **(K)** multiple comparisons test. **, p<0.01; ****, p<0.0001; ns, p>0.05

To determine the impact of transient rest on the epigenome, we used ATAC-seq (27) to analyze differences in chromatin accessibility between Always ON, Always OFF, Rested_7-15_ and Rested_11-15_ HA.28ζ.FKBP CD8+ CAR-T cells (Fig. 5A and fig. S6A). Temporal analyses revealed that T cells experiencing continuous tonic CAR signaling (Always ON) were epigenetically distinct from activated CD19.28ζ CAR-T cells (fig. S6, B and C) and displayed dramatic alterations in chromatin accessibility within the first 7 days (~48,000 peak changes), with fewer changes occurring between D7-11 (~2,300 peak changes) and D11-15 (~2,000 peak changes) (Fig. 5B and fig. S6D). Notably, a 4-day rest period between D7-11 (Rested_D7-11_) or D11-15 (Rested_D11-15_) was associated with ~19,000 and ~15,500 peak changes, respectively (Fig. 5B), many of which were differentially accessible compared to Always ON cells (fig. S6E). These included exhaustion-associated genes *ENTP1* and *BATF*, and the stemness-associated gene *TCF7* (Fig. 5C). These results align with our observations of rapid phenotypic, functional and transcriptional reprogramming following 4 days of rest.

Unbiased Pearson correlation and PCA analyses indicated that rest resulted in global, wholescale remodeling of the epigenome. Always ON cells treated with or without αPD-1 cluster together along PC1 on D11 (66.51% variance) and D15 (58% variance) and exhibit highly correlated genomic accessibility profiles (Fig. 5D and fig. S6, F and G). In contrast, Rested CAR-T cells exhibit clear separation from Always ON and cluster together with Always OFF cells along PC1 (Fig. 5D and fig. S6G). Binding motifs for AP-1 family TFs (BATF, JUNB, NFATC2), which promote T cell exhaustion (8, 28-31), were enriched in accessible regions of the Always ON epigenome, but less accessible following rest (Fig. 5E and fig. S6H). TF binding motifs for many genes implicated in T cell memory were exclusively accessible in rested conditions (TCF7/TCF7L2, LEF1, RUNX family, FOXO family) (23, 32-35), whereas genes associated with T cell exhaustion (EOMES, TBX21) showed exaggerated inaccessibility compared to Always OFF cells (Fig. 5E), raising the prospect that T cell rest could induce a distinct program to drive the development of memory-like T cells. Biological processes inferred from D15 differentially accessible peaks revealed that rest is associated with telomere packaging and G1 phase, diminished Akt signaling, apoptosis, and negative regulation of Wnt signaling (fig. S6H), consistent with induction of a quiescent/memory T cell phenotype.

The epigenetic modifier, enhancer of zeste homolog 2 (EZH2), which catalyzes trimethylation on histone 3 at lysine 27 (H3K27me3) as part of the polycomb repressive complex 2 (PRC2), prevents hematopoietic stem cell exhaustion and is critical for T cell differentiation, maintenance of T cell memory, and antitumor immunity (36, 37). Unbiased PCA of H3K27me3 chromatin immunoprecipitation sequencing (ChIP-seq) separated Always ON and Always OFF or Rested_D11-15_ CAR-T cells along PC2 (Fig. 5F), similar to RNA- and ATAC-seq data (Figs. 3G and 5D), indicating altered H3K27me3 in exhaustion. To test whether EZH2 contributes to reversal of exhaustion in this model, we rested Always ON exhausted CAR-T cells in the presence of the selective EZH2 inhibitor tazemetostat (EZH2i) from D11-15. As expected, EZH2_i_ treatment reduced H3K27me3 levels in all conditions, but preferentially altered the H3K27me3 landscape in Rested_D11-15_ cells (Fig. 5, F and G, fig. S7A). Genomic regions containing decreased H3K27me3 levels in EZH2_i_-treated Rested_D11-15_ cells occurred near or within exhaustion-associated genes that undergo changes in accessibility during rest, including *TBX21, NFATC1*, AP-1 family TFs (*FOS, FOSB, JUNB*), as well as other known regulators of the epigenetic state of exhausted T cells (*DMNT3A and NR4A family TFs*) (Fig. 5H and fig. S7B).

In contrast to inhibitors of DNA methyltransferases and histone acetyltransferases, which strongly attenuated anti-tumor function in all conditions, EZH2_i_ induced only small but detectable effects on Always OFF T cell functionality (fig. S7C) while strongly and dose-dependently attenuating rescue of IL-2 secretion and tumor killing in Rested_D11-15_ CAR-T cells compared to vehicle-treated control. These results demonstrate a requirement for active chromatin remodeling during rest-associated reinvigoration of exhausted CAR-T cells (Fig. 5, I through K). Notably, EZH2_i_ did not affect CAR-T cell viability, proliferation, or other functions (fig. S7, D through H), nor did it alter rest-associated reversal of the cell surface phenotype (fig. S7I), consistent with the observation that the epigenetic mechanisms governing exhausted T cell phenotype and dysfunction are distinct (38). Collectively, these data suggest that CAR-T cell rest remodels the exhaustion-associated epigenome through EZH2, thereby promoting functional reinvigoration.

### Reinvigoration of exhausted CAR-T cells using the Src kinase inhibitor dasatinib

**Figure 6:**
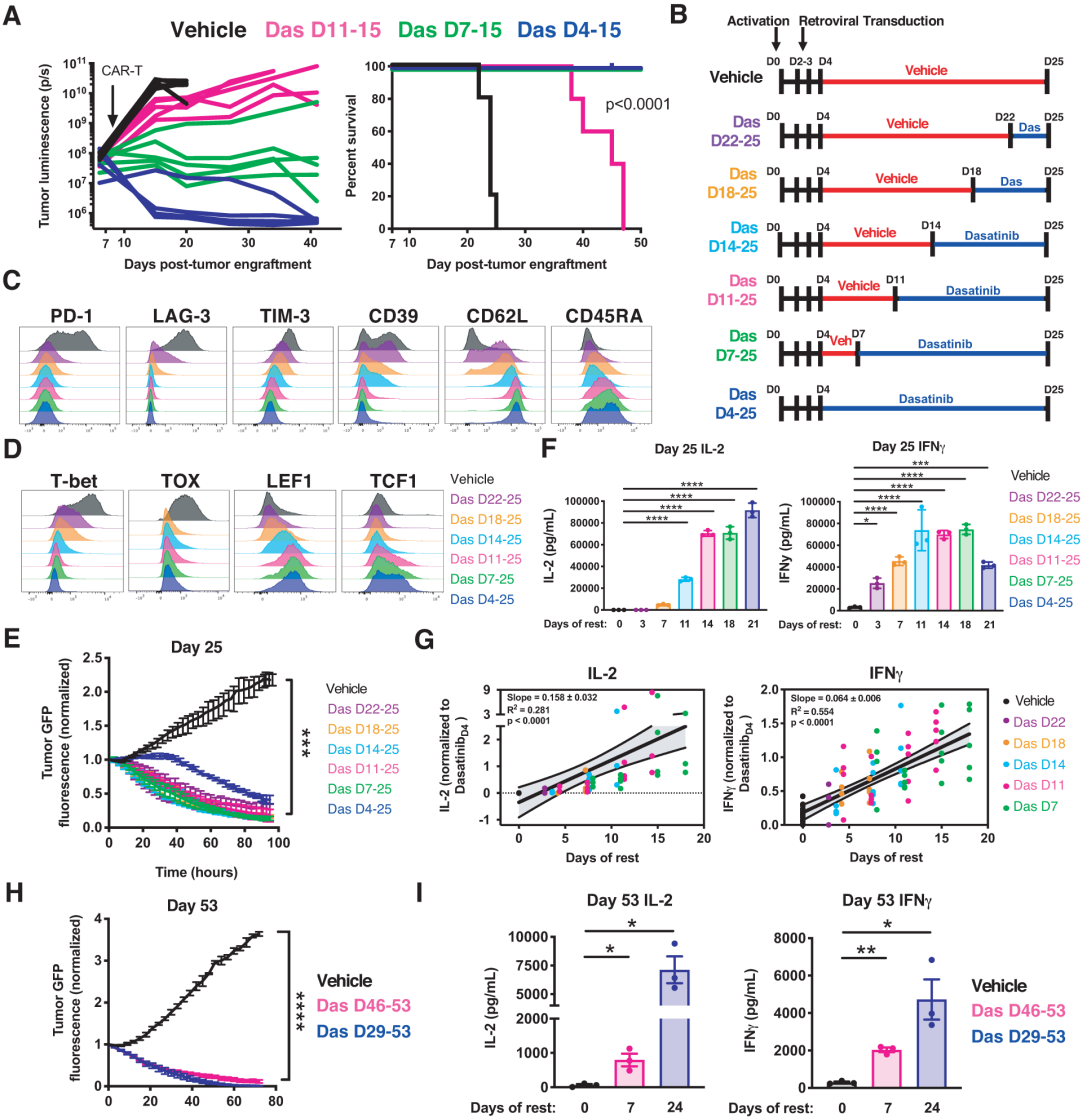
Reinvigoration of exhausted CAR-T cells using the Src kinase inhibitor dasatinib. **A)** Mice engrafted with Nalm6-GD2 were treated with D15 HA.28ζ CAR-T cells expanded in vitro in vehicle or dasatinib (4-11 days). Bioluminescent imaging of tumor growth (left) and survival curves (right) (p<0.0001 log-rank Mantel-Cox test) from a representative experiment (3 individual experiments, n=5 mice/group). **B)** HA.28ζ CAR-T cells were cultured with vehicle (red) or dasatinib (blue) for 3-21 days and collected on indicated days for further analysis. **C-D)** D25 flow cytometric analysis of (C) exhaustion and memory markers and (D) exhaustion- and stemness-associated transcription factors. Representative donor of 3 donors. **E)** Cytotoxicity of D25 CAR-T cells co-cultured with Nalm6-GD2 leukemia (1:4 E:T, normalized to t=0). **F)** D25 IL-2 and IFNγ secretion in response to Nalm6-GD2 leukemia. **G)** D15, D18, D22, and D25 IL-2 and IFNγ secretion levels in response to Nalm6-GD2 leukemia normalized to Dasatinib_D4-25_ for each individual timepoint and plotted based on the number of days of dasatinib treatment. Graphs display data from 4-5 donors (IL-2, n=63 data points. IFNγ, n=85 data points) and a simple linear regression with 95% confidence intervals. D15 IL-2 and IFNγ data from one donor was not assessed. D18 IL-2 from one donor was excluded due to technical artifacts. **H-I)** HA.28ζ CAR-T cells were cultured with vehicle or dasatinib for 7 or 24 days starting on D46 or D29, respectively. (H) Cytotoxic activity of D53 CAR-T cells co-cultured with 143B-GL (1:2 E:T, normalized to t=0). (I) D53 IL-2 and IFNγ secretion in response to Nalm6-GD2 leukemia (mean ± SEM of 3 donors). In B-I Dasatinib was removed 16 hours prior to tumor challenge. In E, F and H error bars represent mean ± SD of triplicate wells from 1 representative donor (n=3 donors). Statistics: **E (**last timepoint) one-way ANOVA and Bonferroni multiple comparisons test, **I** paired or **H** (last timepoint) unpaired two-tailed student’s t test. *, p<0.05; **, p<0.01; ***, p<0.001; ****, p<0.0001; ns, p>0.05

We and others recently demonstrated that dasatinib (Das), an FDA-approved tyrosine kinase inhibitor, suppresses CAR-T cell activation via rapid and reversible antagonism of proximal T cell receptor (TCR) signaling kinases (14, 15). Consistent with this, dasatinib-treated HA.28ζ CAR-T cells exhibited undetectable phosphorylation of CAR CD3ζ and ERK1/2 compared to those treated with vehicle (Fig. S8A). We hypothesized that dasatinib-mediated inhibition of tonic CAR signaling could induce rest and reverse exhaustion. Indeed, HA.28ζ CAR-T cells treated with dasatinib exhibited improved expansion, diminished inhibitory receptor expression, functional reinvigoration (fig. S8, B through D), and improved tumor control following adoptive transfer (Fig. 6A). Ex vivo dasatinib treatment of CAR-T cells expressing GD2.BBζ, which exhibits a lesser degree of tonic signaling (7), also promoted a more memory-like phenotype and enhanced in vivo functionality (fig. S8, E and F), indicating that dasatinib provides an approach to mitigate deleterious CAR signaling in pre-clinical or clinical CAR-T cell manufacturing settings.

We observed in these experiments that Das_D11-15_ T cells did not demonstrate equivalent anti-tumor functionality to Das_D4-15_ and Das_D7-15_ cells. To determine whether Das_D11-15_ cells encountered a point of irreversibility in this model system, or whether this reflected an inadequate period of rest to restore functionality, we interrogated exhaustion reversibility using a more protracted in vitro time course, resting in increments of 3-4 days from D4 until D25 (Fig. 6B). Since dasatinib completely suppresses CAR-T cell signaling and function (fig. S8A) (14, 15), whereas DD-CARs in the OFF state exhibited some leakiness in expression and function (Fig. 1 and fig. S1B), we opted to utilize dasatinib to induce rest in the protracted model system. All groups of D25 dasatinib-treated HA.28ζ CAR-T cells demonstrated diminished exhaustion marker expression and increased stem cell memory-associated CD62L and CD45RA expression (Fig. 6C and fig. S8G), diminished expression of exhaustion associated TFs T-bet and TOX, and increased expression of stemness-associated TFs LEF1 and TCF1 (Fig. 6D and fig. S8H), corroborating D15 epigenetic changes (Fig. 5, C and E). Dasatinib also rescued CAR-T cell cytotoxicity independent of treatment duration (Fig. 5E); however, complete rescue of antigen-induced cytokine secretion only occurred in groups with more prolonged dasatinib exposure (Figure 6F), consistent with a model wherein the incomplete reinvigoration observed on D15 (Fig. 6A and fig. S8D) reflected an insufficient period of rest rather than irreversibility of the exhaustion program (Fig. 6, F through G). This model is further corroborated by the correlation observed between the degree of functional reinvigoration and duration of rest, which was independent of the time at which rest was initiated (Fig. 6G and fig. S8, I through K). Initiation of rest at D29 or as late as D46 induced partial reversal of phenotypic and functional exhaustion hallmarks on D53, and was associated with decreased T-bet and TOX expression, indicating marked plasticity in the exhaustion program even at very late time points (Fig. 6, H and I, and fig. S9).

### Intermittent CAR-T cell rest mitigates exhaustion and enhances anti-tumor functionality

Adoptive transfer of rested CAR-T cells induced more durable anti-tumor responses than exhausted CAR-T cells (Figs. 4I and 6A), suggesting that rested cells might resist reacquisition of the exhaustion program upon reactivation. To test this hypothesis, HA.28ζ exhausted CAR-T cells were “pulsed” with dasatinib for 4 days, then dasatinib was removed for 3 days prior to analysis (fig. S10A). CAR-T cells pulsed with dasatinib exhibited improved CD8+ expansion, diminished exhaustion marker expression, and enhanced functionality compared to HA.28ζ CAR-T cells treated with vehicle (fig. S10, B through E). Similar results were observed by toggling HA.28ζ.FKBP CAR surface expression (fig. S10, F through J), suggesting that repeated rest periods may prolong responses in vivo.

**Figure 7:**
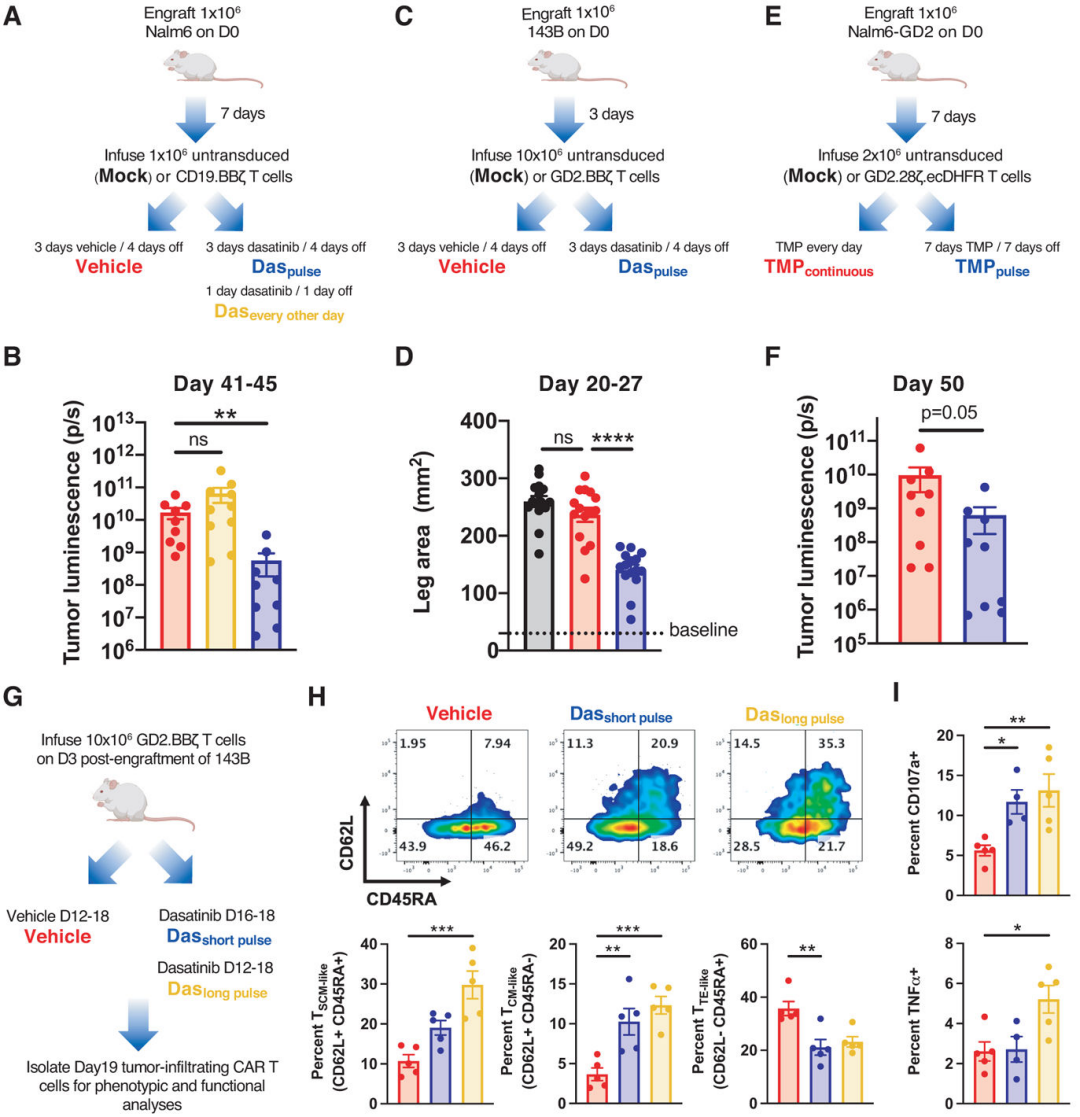
Intermittent CAR-T cell rest mitigates exhaustion and prolongs anti-tumor responses in vivo. **A-B**) Mice engrafted with Nalm6 leukemia and treated with CD19.BBζ CAR-T cells were (A) dosed with vehicle (red), dasatinib twice daily in a 3 day on/4 day off pulsed schedule (blue), or dasatinib every other day from D11-21 (green) (12 total doses/mouse for each condition). (B) D41-45 bioluminescence imaging of tumor growth (2 individual experiments, n=9-10 mice/group). **C-D)** Mice engrafted with 143B-GL osteosarcoma cells and treated with GD2.BBζ CAR-T cells were (C) dosed with vehicle (red) or dasatinib twice daily in a 3 day on/4 day off pulsed schedule (blue). (D) D20-27 caliper measurement of tumor growth (3 individual experiments, n=15 mice/group) **E-F)** Mice engrafted with Nalm6-GD2 leukemia and treated with GD2.28ζ.ecDHFR CAR-T cells were (E) dosed with TMP continuously (red) or pulsed in a 7 days TMP/7 days OFF schedule (blue). (F) D50 bioluminescence imaging of tumor growth (2 independent experiments, n=10 mice/group from; 1 mouse from each group did not survive until D50 and were thus excluded). **G-I)** Mice engrafted with 143B-GL osteosarcoma cells and treated with GD2.BBζ CAR-T cells were (G) dosed with vehicle (red) or dasatinib twice daily from D12-18 (green) or D16-18 (blue). Tumor-infiltrating CD8+ CAR-T cells (CAR-TIL) were isolated on D19 and analyzed by flow cytometry for (H) effector and stem memory markers and (I) for degranulation (CD107a+) and TNFα after 6-hour ex vivo stimulation with Nalm6-GD2. Statistics: **B and H** (Percent T_TE-like_) Kruskal-Wallis test and Dunn’s multiple comparisons test, **D, H** (Percent T_SCM-like_ and T_CM-like_), and **I** one-way ANOVA and Dunnett’s multiple comparisons test, **F** Mann-Whitney test. *, p<0.05; **, p<0.01; ***, p<0.001; ****, p<0.0001 ns, p>0.05

To test whether intermittent rest results in more durable anti-tumor responses, mice engrafted with dasatinib-insensitive liquid (Nalm6 leukemia) (14) or solid (143B osteosarcoma) (39) tumors were infused with CAR-T cells and repeatedly pulsed with dasatinib or vehicle. Nalm6-bearing mice infused with a limiting dose of CD19.BBζ CAR-T cells and pulsed with dasatinib using a 3-day dasatinib/4-day off schedule exhibited more durable anti-tumor responses and improved survival compared to mice treated with vehicle or dasatinib every other day (Fig. 7, A and B, and fig. S11, A and B), further corroborating in vitro data showing that the duration of rest correlates with the degree of exhaustion reversal (Fig. 4C and Fig. 6, A through G). Similar results were achieved in 143B-bearing mice infused with GD2.BBζ CAR-T cells, wherein repeated 3-day dasatinib pulses slowed tumor growth and enhanced survival (Fig. 7, C and D, and fig. S11, C and D), and in Nalm6-GD2-bearing mice infused with drug-regulatable GD2.28ζ.ecDHFR CAR-T cells, wherein toggling CAR expression using a 1-week TMP/1-week OFF dosing schedule improved tumor control (Fig. 7, E and F).

To confirm that rest mediated CAR-T cell exhaustion reversal in vivo, tumor bearing GD2.BBζ CAR-T treated mice were treated with dasatinib for 3 or 7 consecutive days starting at D16 or D12 post-engraftment (Fig. 7G), time points associated with onset of CAR-T cell exhaustion (8) and failure to control tumor growth (fig. S11E). Tumor-infiltrating CAR-T cells (CAR-TIL) on D19 exhibited diminished inhibitory receptor expression, increased frequencies of memory-like cells, and augmented functionality (Fig. 7, H and I, and fig. S11, F and G), indicating that transient cessation of antigen-induced CAR signaling in vivo reverses hallmarks of exhaustion. Collectively, these results demonstrate that pharmacologic antagonism of TCR signaling kinases prevents or reverses CAR-T cell exhaustion and intermittent CAR-T cell rest results in superior antitumor responses in vivo.

### DISCUSSION

Chronic antigen stimulation induces T cell exhaustion, which is associated with a heritable epigenetic imprint distinct from effector and memory T cells (16, 17, 40-42). Therapeutic agents targeting the PD-1/PD-L1 axis can reinvigorate exhausted T cells (43), but do not reverse the exhaustion-associated epigenetic imprint (16). Recent studies have identified TOX, TOX2, and AP-1 family members as central regulators of T cell exhaustion that promote widespread transcriptional and epigenetic dysregulation (38, 44-46), enabling new approaches to mitigate exhaustion, including enforced expression of c-Jun (S) or CRISPR-mediated deletion of TOX, TOX2, or NR4A family TFs (44, 47). However, such modifications have not reversed the exhaustion-associated epigenetic imprint, leading many to suggest that the imprint is fixed.

In this study, we modified a validated in vitro model of T cell exhaustion, wherein tonic CAR signaling induces hallmark phenotypic, functional, transcriptomic and epigenetic features of exhaustion within 11 days (HA.28ζ) (8), to enable precise, drug-dependent control of CAR signaling. Consistent with murine models of viral infection wherein antigen clearance induces T cell memory rather than exhaustion (9, 21, 22, 41, 48), early cessation of CAR signaling (Rested_D7-11_) redirected T cell differentiation away from exhaustion and toward a memory-like state. When inhibition of CAR signaling was delayed until D11, after cells had already acquired hallmark features of T cell exhaustion, we observed impressive functional reinvigoration associated with global phenotypic, transcriptomic and epigenetic reprogramming. Similar results were obtained using the tyrosine kinase inhibitor dasatinib to inhibit CAR signaling, where functional reinvigoration was observed even in CAR-T cells subjected to prolonged antigen-independent tonic signaling or antigen-induced signaling in vivo.

Several groups have sought to identify subsets of exhausted T cells with capacity for reversal of the exhaustion program (24, 25, 49, 50), leading to the discovery of “progenitor exhausted” T cells, which express the stemness transcription factor TCF1 (gene name *Tcf7*) and exhibit increased accessibility at the *Tcf7* locus (17). Progenitor exhausted cells exhibit greater reprogrammability and are apparently essential for the proliferative burst following PD-1 blockade (23-26, 49). One model to explain our findings posits that cessation of tonic CAR signaling induces preferential expansion of progenitor exhausted T cells. Consistent with this, rested CAR-T cell populations exhibited increased accessibility at the *Tcf7* locus, enriched *Tcf7* binding motifs within accessible regions of the genome, and increased frequency of TCF1+ cells compared to exhausted CAR-T cells. However, TCF1+ cells in reinvigorated dasatinib-treated groups did not co-express PD-1 or other immune checkpoint receptors, a canonical feature of progenitor exhausted T cells (23-26, 49). Further, clonotypic analyses demonstrated similarly high levels of TCR diversity in exhausted and rested cell populations, indicating that transcriptional and epigenetic alterations induced by rest in this model system did not involve preferential expansion of a small subset of clones.

An alternative model that is most consistent with the data presented here posits that exhausted cell populations that have acquired the hallmark epigenetic imprint retain the capacity for epigenetic remodeling to resemble healthy, non-tonically signaling CAR-T cells. Results demonstrating that tazemetostat, an EZH2 inhibitor, prevents complete functional reinvigoration induced by rest in this model system are consistent with epigenetic remodeling rather than enrichment of progenitor exhausted cells. Future studies are warranted to better define the specific cell populations that undergo epigenetic remodeling following T cell rest and identify the precise role of EZH2 in remodeling of the exhaustion-associated epigenome.

Irrespective of mechanism, these results demonstrate that cessation of CAR signaling augments function in cells transitioning to exhaustion and in those already endowed with hallmark features of exhaustion, and is distinct from that which is induced by PD-1/PD-L1 blockade. Further, dasatinib promoted T cell memory during ex vivo expansion, suggesting that incorporation of dasatinib during manufacturing of clinical CAR-T cell products could enhance efficacy following adoptive transfer, which is consistent with studies showing that ex vivo kinase inhibitor treatment improves T cell functionality (51-54).

Our work also demonstrates that intermittent CAR signaling in vivo can prevent or reverse exhaustion and thereby enhance anti-tumor responses in liquid and solid tumor models independent of the CAR costimulatory domain (CD28 versus 4-1BB) or the propensity for tonic CAR signaling (CD19 binder versus GD2 binder). The findings raise the prospect that regulatable CAR platforms developed to mitigate CAR-mediated toxicity (18, 55-59), including the DD-CAR system described here, may also enhance CAR-T cell efficacy as a result of temporal control of CAR-T cell signaling. Consistent with this, a recent study testing a regulatable CD19-targeting CAR demonstrated that CAR-T cells provided a longer rest phase exhibited superior antigen-induced expansion compared to those that received rest for a shorter period (60). Additional studies are needed to determine whether this approach can be universally applied to all CARs and whether remodeling of the exhaustion-associated epigenome is the mechanism by which rest augments functionality in non-tonic signaling CAR-expressing T cells in vivo. Of note, the observation that intermittent rest augments functionality is arguably paradoxical, since CAR-T cell inactivation would be expected to provide periods of unopposed tumor growth and thereby reduce efficacy. Ultimately, maneuvers designed to induce “rest” in the context of cancer immunotherapy are likely to be more effective if approaches are developed that enable phased periods of rest to a portion of the T cell population rather than resting the entire population en masse, and thereby maintaining consistent anti-tumor immune pressure.

The findings presented here also raise the prospect that therapies designed to transiently inhibit TCR signaling might enhance functionality of exhausted, non-engineered T cell populations. This hypothesis has been tested to a limited extent in murine models of chronic virus or cancer, whereby removal of antigen failed to reinvigorate exhausted T cells (9, 17, 50, 61). Discrepancies between our observations and those in murine models could be explained by intrinsic differences in murine and human exhausted T cells’ potential for functional reinvigoration. Indeed, improved antigen-specific T cell function has been associated with antigen clearance in humans with hepatitis C infection treated with direct acting anti-viral therapies (62-66). Similarly, an immunomodulatory effect of dasatinib on T cells has been associated with improved anti-tumor immunity (67, 68), and BRAF/MEK inhibition in patients with melanoma leads to upregulation of *TCF7* and expansion of melanoma-specific TIL (69). Collectively, these observations suggest that transient cessation of TCR signaling could provide a widely applicable but underappreciated approach to enhance functionality in populations of exhausted human T cells; however, additional studies are needed to more fully define the effects of rest on non-engineered, exhausted T cells.

In summary, we demonstrate that transient cessation of CAR signaling can restore functionality and induce epigenetic reprogramming in exhausted human CAR-T cell populations. These results suggest that CAR-T cell therapeutics designed to incorporate periods of rest may exhibit superior efficacy compared to constitutive platforms, and raise the prospect that targeting of proximal TCR/CAR signaling kinases may represent an immunotherapeutic strategy for mitigating T cell exhaustion.

## Supplementary Material

Weber_Science2021_supplementary
