# Supplementary material for: Transient “rest” restores functionality in exhausted CAR-T cells via epigenetic remodeling
Source: Science. Author manuscript; Available in PMC 2021 Apr 15. (PMC8049103; doi:10.1126/science.aba1786)
Supplement: Weber_Science2021_supplementary [file NIHMS1682316-supplement-Weber_Science2021_supplementary.pdf]

Supplementary Materials for

Transient "rest" restores functionality in exhausted CAR-T cells via epigenetic remodeling

Evan W. Weber, Kevin R. Parker^, Elena Sotillo^, Rachel C. Lynn, Hima Anbunathan, John Lattin, Zinaida Good, Julia A. Belk, Bence Daniel, Dorota Klysz, Meena Malipatlolla, Peng Xu, Malek Bashti, Sabine Heitzeneder, Louai Labanieh, Panayiotis Vandris, Robbie G. Majzner, Yanyan Qi, Katalin Sandor, Ling-Chun Chen, Snehit Prabhu, Andrew J. Gentles, Thomas J. Wandless, Ansuman T. Satpathy, Howard Y. Chang, Crystal L. Mackall*

^These authors contributed equally to this work

*corresponding author: cmackall@stanford.edu

This PDF file includes:

Materials and Methods
Figs. S1 to S11
Table S1

Other Supplementary Materials for this manuscript includes the following:

N/A

MATERIALS AND METHODS

Primary human T cells

Anonymous healthy donor buffy coats were obtained from the Stanford University Blood Center (Stanford, CA) under a University Institutional Review Board-exempt protocol. Written informed consent was obtained from all healthy donors. Primary CD3+ T cells were subsequently isolated from buffy coats via negative selection (described below).

Cell Lines

143B osteosarcoma cells (American Type Culture Collection, ATCC) were stably transduced to express GFP and firefly luciferase (143B-GL). Nalm6 B-ALL cell line that was stably transduced to express GFP and firefly luciferase (Nalm6-GL). Nalm6-GL was co-transduced with cDNAs for GD2 synthase and GD3 synthase in order to induce overexpression of GD2 (Nalm6-GD2). Single cell clones were then chosen for high antigen expression. All cell lines were cultured in complete medium (RPMI supplemented with 10% FBS, 10 mM HEPES, 2mM GlutaMAX, 100 U/mL penicillin, and 100 μg/mL streptomycin (Gibco).

Murine xenograft models

NOD/SCID/IL2R$\gamma^{-/-}$ (NSG) mice were bred, housed, and treated under Stanford University Administrative Panel on Laboratory Animal Care (APLAC)-approved protocols. 6-8 week old male or female mice used in experiments were healthy, drug and test naïve, and were not involved in previous procedures. Mice were housed in sterile cages in the barrier

facility located in the Veterinary Service Center (VSC) at Stanford University with a 12-hour light/dark cycle. Mice were monitored daily by the VSC husbandry staff and were euthanized upon manifestation of hunched posture, impaired mobility, rough coat, paralysis, or tumor sizes that exceeded 17 cm in length or in width.

For xenograft experiments using Nalm6-GD2, $1x10^6$ tumor cells in 200 μL PBS were engrafted via intravenous injection. $1\text{-}2x10^6$ CAR-T cells suspended in 200 μL PBS were subsequently infused intravenously on day 7 post-tumor injection. Tumor burden was monitored via bioluminescence imaging using a Spectrum IVIS instrument and quantified using Living Image software (Perkin Elmer).

For xenograft experiments using 143B cells, $1x10^6$ tumor cells in 100 μL PBS were engrafted via intramuscular injection into the flank and $10x10^6$ CAR-T cells suspended in 100 μL PBS were infused intravenously on day 3 post-injection. Tumor size was monitored via caliper measurements. Mice were randomized to ensure equal tumor burden before CAR-T cell treatment.

For experiments involving GD2.28ζ.ecDHFR or HA.28ζ.ecDHFR-expressing CAR-T cells, mice received 1) 200 μL of vehicle (ddH_2O, Thermofisher, # 10-977-023) injected intraperitoneally once daily for 6-7 days/week, or 2) 200 μL of 200 mg/kg trimethoprim lactate (TMP, Bioworld, #42010018-3) resuspended in ddH_2O injected intraperitoneally

once daily for 6-7 days/week, or 3) 200 μL of 100 mg/kg trimethoprim lactate resuspended in a 75:25 mixture of ddH_2O/polyethylene glycol (Sigma-Aldrich, #202398) administered orally twice daily for 6-7 days/week.

For experiments involving CD19.BBζ and GD2.BBζ-expressing CAR-T cells, mice received either 1) 200 μL of vehicle (40:60 H_2O/polyethylene glycol), or 2) 200 μL of 50 mg/kg dasatinib (Eton Bioscience, #1100140103) resuspended in vehicle, which were administered orally twice daily for 7 days/week as previously described (*14*).

CAR construct design

CD19.BBζ, GD2.BBζ, and HA.28ζ CARs (*7, 8*) were constructed in MSGV retroviral vectors. GD2.28ζ.DD and HA.28ζ.DD CARs were constructed in pELNS lentiviral vectors. Codon-optimized gene fragments that included a portion of the CAR CD3z domain sequence (downstream of the BmgBI restriction site) and FKBP12 or ecDHFR destabilizing domains were generated by IDT. Fragments were then inserted into pELNS.GD2.28ζ and pELNS.HA.28ζ vectors at the 5' BmgBI restriction cut site within the CAR CD3z domain and the 3' SalI site downstream of the stop codon using T4 DNA Ligase (New England Biolabs, #M0202L), which enabled insertion of the DD without introduction of any additional nucleotides or linker sequences. Plasmids were amplified by transforming XL-10 gold ultracompetent cells per the manufacturer's protocol (Agilent Technologies, #200315), and final sequences were validated via DNA sequencing from Elim Biopharmaceuticals.

Virus production

For third generation, self-inactivating lentiviral production, 15 million 293T cells were plated on a 15 cm dish for 24 hours prior to transfection. On the day of transfection, 120 μL of lipofectamine 2000 (Thermofisher, #11668500) was mixed with 1.5 mL of room temperature Opti MEM (Fisher Scientific, #31-985-088) for 5 minutes. A mixture of 18 μg pRSV-Rev plasmid, 18 μg pMDLg/pRRe (Gag/Pol), 7 μg pMD2.G (VSVG envelope), and 15 μg pELNS vector plasmid in 1.5mL Opti MEM were added in dropwise fashion to the lipofectamine mixture and allowed to incubate for 30 minutes at room temperature. The 3 mL transfection mixture was then added to 17 mL of 293T complete medium (DMEM, 10% FBS, 1% penicillin/streptomycin/glutamine), which was then applied to 293T cell plates. At 24- and 48-hours post-transfection, supernatant was collected, centrifuged at 500 g, passed through at 0.45 μm syringe filter (Millipore, #SLHV033RB), and frozen at -80 C. Retrovirus was produced in the 293GP packaging cell line as previously described (*7*). Briefly, 70% confluent 293GP 15 cm plates were co-transfected with 20ug MSGV vector plasmid and 10 μg RD114 envelope plasmid with lipofectamine 2000. Viral supernatants were collected at 48- and 72-hours post-transfection, centrifuged to separate cell debris from the viral supernatant, and frozen at -80 C for future use.

T cell isolation

Primary human T cells were isolated from healthy donor buffy coats using the RosetteSep Human T cell Enrichment kit (Stem Cell Technologies), Lymphoprep density gradient

medium, and SepMate-50 tubes according to the manufacturer's protocol. Isolated T cells were cryopreserved at 1-2x10^7 T cells per vial in CryoStor CS10 cryopreservation medium (Stem Cell Technologies).

Lentiviral and retroviral transduction

Human T cells were transduced with lentivirus by adding thawed lentiviral supernatant directly onto 0.5-1x10^6 T cells plated in 12- or 24-well plates. For retroviral transduction, non-tissue culture treated 12- or 24-well plates were coated overnight with 1 mL or 0.5 mL of 25 μg/mL retronectin in PBS (Takara), respectively. Plates were washed 2X with PBS and blocked with 2% BSA + PBS for 15 minutes. Thawed retroviral supernatant was added at ~1 mL per well and centrifuged at 3200 RPM for 1.5-2 hours at 32 C before the addition of 0.5-1x10^6 T cells.

CAR-T cell culture

Cryopreserved T cells were thawed and activated on day 0 with Human T-Expander αCD3/CD28 Dynabeads (Gibco) at 3:1 bead:cell ratio in T cell medium (AIMV supplemented with 5% FBS, 10 mM HEPES, 2 mM GlutaMAX, 100 U/mL penicillin, and 100 μg/mL streptomycin (Gibco). Recombinant human IL-2 (Peprotech) was provided at 100 U/mL. Lentiviral transduction was done on day 1 and retroviral transductions were done on days 2 and 3 post-bead activation. Magnetic beads were removed on day 4, and T cells were cultured with fresh medium every 2-3 days and normalized to 0.5 x 10^6 cells/mL. For culture conditions requiring αPD-1, 5 μg/mL nivolumab (Selleck Chemicals, A2002)

was added on D7 and supplemented every 2-3 days. For culture conditions requiring shield-1 (1 μM unless otherwise noted), trimethoprim (1 μM unless otherwise noted), dasatinib (1 μM), tazemetostat (1 μM), decitabine (1 μM) (Sigma-Aldrich, A3656), or entinostat (1μM) (Selleck Chemicals, S1053), freshly thawed drug was mixed with T cell culture medium and supplemented every 2-3d days. HA.28ζ.FKBP CAR-T cells were rested via removal of either shield-1 or trimethoprim from the culture medium, whereby cells were washed 2X in PBS and new conicals were used for each wash ensure complete drug washout, and subsequently resuspended in fresh T cell culture medium lacking shield-1/trimethoprim.

Immunoblotting

Whole-cell protein lysates were obtained in non-denaturing buffer (150 mmol/L NaCl, 50 mmol/L Tris-pH8, 1% NP-10, 0.25% sodium deoxycholate). Protein concentrations were estimated by using with DC Protein colorimetric assay (BioRad, 5000116). Per sample, 20 μg of protein was mixed with 5X reducing loading buffer (Pierce, 39000), boiled at 95 C for 5 min and loaded onto 11% PAGE gels. After electrophoresis, protein was transferred to PVF membranes. Signals were detected by enhanced chemiluminescence (Pierce) or with the Odyssey imaging system. Representative blots are shown. The following primary antibodies used were purchased from Cell Signaling: total ERK1/2 (no. 9102) and Phospho-ERK1/2 (no. 9101). The CD3ζ (4A12-F6) and phospho-CD3ζ (EP265(2)Y) antibodies were purchased from Abcam.

Cytokine secretion assays

$5x10^4$ CAR-T cells and $5x10^4$ tumor cells were cultured in 200 μL T cell medium (no supplemented IL-2) with or without shield-1/trimethoprim in 96-well flat bottom plates for 24 hours. For 1A7 anti-idiotype stimulation, serial dilutions of 1A7 were immobilized to Nunc Maxisorp 96-well ELISA plates (Thermo Scientific) in 1X Coating Buffer (BioLegend) overnight at 4 C. Wells were washed once with PBS and $1x10^5$ CAR-T cells were plated in 200 μL T cell medium (no supplemented IL-2) and cultured for 24 hours. Triplicate wells were plated for each condition. In some conditions, stabilizing drug (shield-1 or trimethoprim) was added to DD-CAR-T cells in order to normalize CAR expression across experimental conditions. Culture supernatants were collected and analyzed for IL-2 and IFNy concentration via ELISA using the manufacturer's protocol (BioLegend).

Incucyte killing assay

$5x10^4$ GFP+ tumor cells were co-cultured with CAR-T cells in triplicate at a 1:1, 1:2, 1:4, or 1:8 Effector:Target ratio in 200 μL T cell medium lacking IL-2 in 96-well flat bottom plates. Plates were imaged every 2-6 hours for up to 120 hours using the Incucyte ZOOM Live-Cell analysis system (Essen Bioscience). 4 images per well at 10X zoom were collected at each time point. Total integrated GFP intensity per well or total GFP area (μm^2/well) was assessed as a quantitative measure of live, GFP+ Nalm6-GD2 or GFP+ 143B-GL tumor cells, respectively. Values were normalized to the t=0 measurement. Effector:Target (E:T) ratios are indicated in the figure legends.

Flow cytometry and cell sorting/purification

CAR-T cells were washed 2X in FACS buffer (2% FBS + PBS) prior to cell surface staining with fluorescent-labeled antibodies. Cells were resuspended in 100 μL of antibody mastermix and incubated at 4 C for 30 minutes. Cells were washed 2X in FACS buffer and analyzed on a BD Fortessa running FACS Diva software or sorted at the Stanford Shared FACS Facility on a BD FACSAria running FACS Diva software. For experiments involving multiple donors analyzed on different days, saved application settings were used to enable meaningful comparisons between donors. For experiments involving staining of intracellular transcription factors, cells were surface stained as described above, then fixed, permeabilized and labeled according to the FoxP3 Transcription Factor Staining Buffer Set manufacturer's protocol (eBioscience, # 00-5523-00). For experiments involving CellTrace Violet (Thermo Fisher, #C34557), CAR-T cells were loaded with 5 μM dye + PBS for 10 minutes at 37 C per the manufacturer's protocol. The 1A7 anti-14G2a idiotype antibody used to detect surface CAR was conjugated in-house with Dylight488 and/or 650 antibody labeling kits (Thermo Fisher). In some ATAC-seq experiments (fig. S6, B and C) and in all ChIP-seq experiments, CD8+ T cells were isolated via Miltenyi negative selection using anti-CD4 magnetic selection beads and following the manufacturer's protocol.

Intracellular cytokine staining

CAR-T cells and 143B-GL were plated at a 1:1 E:T ratio in T cell medium (no supplemented IL-2) containing 1X monensin (eBioscience) and 1 μL/test CD107a antibody (Clone H4A3, BioLegend) for 5-6 hours. After incubation, intracellular cytokine staining

was performed using the FoxP3 Transcription Factor Staining Buffer Set according to the manufacturer's protocol.

Murine tissue analyses

To calculate circulating numbers of adoptively transferred human T cells, peripheral blood was sampled from live mice via retro-orbital blood collection under isoflurane anesthesia. 50 μL blood was labeled with anti-CD45 (eBioscience), lysed using BD FACS Lysing Solution, and quantified using CountBright Absolute Counting beads (Thermo Fisher) on a BD Fortessa cytometer. For phenotypic analysis of blood, spleen, and tumors, mice were euthanized and blood was harvested via cardiac puncture. Spleens and tumors were mechanically dissociated and washed 2X in PBS. Both blood and spleen were treated with ACK lysis buffer (Fisher Scientific, # 50-751-7469) for 5 minutes at room temperature., after which the reaction was quenched with FACS buffer. Cell suspensions were washed 2X, stained with anti-human antibodies (as described in "Flow Cytometry") and analyzed on a BD Fortessa cytometer. For experiments involving ex vivo stimulation of tumor-infiltrating CAR-T cells, whole tumor cell suspensions were mixed with $0.5x10^6$ Nalm6-GD2 for 6 hours at 37 C, stained with anti-human antibodies and analyzed on a BD Fortessa cytometer (see Intracellular cytokine staining).

Mass cytometry

1-2 x 10^6 T cells from each culture condition were washed 2X in PBS (Rockland). Cells were resuspended in 1 mL of 250 nM cisplatin + PBS (Fluidigm Catalog: 201064), which was used to assess cell viability. After a 3-minute incubation at room temperature, the

cisplatin reaction was quenched with cell staining medium (CSM, 1X PBS with 0.05% BSA and .02% sodium azide). Cells were washed 2X in PBS and incubated in CSM + anti-CCR7 and anti-CXCR3 antibodies for 30 minutes at room temperature. These cell surface proteins were undetectable when labeled post-fixation, and thus, required labeling at this step. After 2 washes, cells were then fixed with 1.6% paraformaldehyde + PBS for 10 min at room temperature, followed by 2 washes with 1X PBS. Samples were subsequently flash frozen on dry ice for further use. Upon thawing and washing in CSM, barcoding was performed using a palladium-based approach using the Cell-ID 20-Plex Pd Barcoding Kit manufacturer's protocol (Fluidigm Corporation). Cell samples were mixed together and treated as one sample for all subsequent steps. Titrated amounts of each cell surface antibody were mixed, filtered through a 0.1 μm spin filter (Millipore), and added to the merged barcoded sample for 30 minutes at room temperature. Cells were then washed 2X with CSM and permeabilized by adding ice cold methanol for 10 minutes at 4C, followed by two washes with CSM. Finally, samples were resuspended in IR-intercalator (0.5 μM iridium intercalator and 1% PFA in 1X PBS), washed 1X with CSM and 2X with ddH_2O, and filtered through a 50 μm cell strainer (Thermofisher). Cells were then resuspended at $1x10^6$ cells/mL in ddH_2O with 1x EQ four-element beads (Fluidigm Corporation, #201078). Cells were acquired on a Fluidigm Helios mass cytometer.

ATAC-seq

CAR-T cells were FACS sorted on CD8 or selected via Miltenyi isolation (see Cell Sorting) and processed for ATAC-seq, which was carried out as previously reported using freshly

isolated cells or viably frozen cells (in which case, the OMNI-ATAC-seq protocol was used) (*70*). Briefly, 50,000 cells from each sample were centrifuged at 500 g at 4°C, then resuspended in 50 μL of cold ATAC-seq Resuspension Buffer (RSB) (10 mM Tris-HCl, 10 mM NaCl, 3 mM $MgCl_2$) supplemented with 0.1% NP-40,0.1% Tween-20, and 0.01% digitonin. Samples were incubated on ice for 3 minutes, then washed with 1 mL RSB supplemented with 0.1% Tween-20. Nuclei were pelleted at 500 g for 10 minutes at 4°C. The nuclei pellet was resuspended in 50 μL transposition mix (25 μL 2x TD buffer, 2.5 μL transposase (Illumina), 16.5 μL PBS, 0.5 μL 1% digitonin, 0.5 μL 10% Tween-20, 5 μL H_2O) and incubated at 37°C for 30 minutes in a thermomixer set to 1000 RPM. DNA clean-up was achieved using the Zymo DNA Clean and Concentrator-5 Kit (#D4014). Libraries were PCR-amplified using the NEBNext Hi-Fidelity PCR Master Mix and custom primers (IDT) as described previously (*27*). Libraries were sufficiently amplified following 5 cycles of PCR, as indicated by qPCR fluorescence curves (*27*). Libraries were cleaned and purified using the Zymo DNA Clean and Concentrator-5 Kit and subsequently quantified with the KAPA Library Quantification Kit (#KK4854). Libraries were sequenced on the Illumina NextSeq at the Stanford Functional Genomics Facility with paired-end 75 bp reads.

ChIP-seq

ChIP-seq was performed as previously described with minor modifications (*71*). CAR-T cells (3 x 10^6) were double crosslinked by 50 mM DSG (disuccinimidyl glutarate, #C1104 - ProteoChem) for 30 minutes followed by 10 minutes of 1% formaldehyde. Formaldehyde

was quenched by the addition of glycine. Nuclei were isolated with ChIP lysis buffer (1% Triton x-100, 0.1% SDS, 150 mM NaCl, 1 mM EDTA, and 20 mM Tris, pH 8.0). Nuclei were sheared with Covaris sonicator using the following setup: Fill level – 10, Duty Cycle – 5, PIP – 140, Cycles/Burst – 200, Time – 4 minutes). Sheared chromatin was immunoprecipitated with anti-H3K27me3 antibody overnight (C15410069 – Diagenode, 2 μg/sample). Antibody chromatin complexes were pulled down with Protein A magnetic beads and washed once in IP wash buffer I (1% Triton, 0.1% SDS, 150 mM NaCl, 1 mM EDTA, 20 mM Tris, pH 8.0, and 0.1% NaDOC), twice in IP wash buffer II (1% Triton, 0.1% SDS, 500 mM NaCl, 1 mM EDTA, 20 mM Tris, pH 8.0, and 0.1% NaDOC), once in IP wash buffer III (0.25 M LiCl, 0.5% NP-40, 1 mM EDTA, 20 mM Tris, pH 8.0, 0.5% NaDOC) and once in TE buffer (10 mM EDTA and 200 mM Tris, pH 8.0). DNA was eluted from the beads by vigorous shaking for 20 minutes in elution buffer (100 mM $NaHCO_3$, 1% SDS). DNA was decrosslinked overnight at 65C and purified with MinElute PCR purification kit (Qiagen). DNA was quantified by Qubit and 20 ng DNA was used for sequencing library construction with the Ovation Ultralow Library System V2 (Tecan) using 10 PCR cycles. Libraries were sequenced on the Illumina NovaSeq platform using paired-end 75 bp read configuration.

Flow cytometry data analysis

Flow cytometry data were analyzed using FlowJo v10.4.2 software. For analyses of median fluorescent intensities (MFI), background subtraction was performed by subtracting MFI values of unstained, idiotype, or fluorescence minus one (FMO) controls from the MFI of each experimental sample. Due to the bimodal nature of CD39 expression, in some

experiments CD39 MFI was quantified only for the CD39 positive cell subset (represented as "CD39+ MFI").

Mass cytometry data processing and analysis

Acquired data were normalized and debarcoded using MATLAB-based software . FCS files were imported into Cytobank and traditional handing gating was used to select single, viable CAR+/CD8+ T cells. New FCS files were then exported and arcsinh-transformed with a co-factor of 5, after which 2,000 cells were randomly downsampled per FCS file. Additional columns were added to each FCS file representing: day of collection (numeric value 7-11), timepoint (numeric value 1-5), and exhaustion and memory scores. Exhaustion score for each cell was calculated as mean expression of PD-1, TIM-3, LAG-3, 2B4, CTLA-4, BTLA, and CD39 normalized to mean expression of these markers in control HA.28ζ.FKBP T cells cultured in the absence of shield-1 at each time point. Similarly, memory score was calculated using CD45RA, IL-7R, CD27, CD197 markers.

$$\text{Exhaustion Score} = \text{Sample}_{(\text{PD-1 MFI} + \text{TIM-3 MFI} + \text{LAG-3 MFI} + \text{2B4 MFI} + \text{CTLA-4 MFI} + \text{BTLA MFI} + \text{CD39 MFI})} / \text{Always OFF}_{(\text{PD-1 MFI} + \text{TIM-3 MFI} + \text{LAG-3 MFI} + \text{2B4 MFI} + \text{CTLA-4 MFI} + \text{BTLA MFI} + \text{CD39 MFI})}$$

$$\text{Memory Score} = \text{Sample}_{(\text{CD45RA MFI} + \text{IL-7R MFI} + \text{CD27 MFI} + \text{CD197})} / \text{Always OFF}_{(\text{PD-1 MFI} + \text{TIM-3 MFI} + \text{LAG-3 MFI} + \text{2B4 MFI} + \text{CTLA-4 MFI} + \text{BTLA MFI} + \text{CD39 MFI})}$$

Sampled/edited FCS files from days 7-11 for each condition were concatenated, creating an Always ON and Rested$_{D7\text{-}11}$ files each with 10,000 total events. To create force-directed layouts (FDLs), we used *Vortex* software (*20*). Edges, a basis for spring-like attractive forces between single cells within the FDL, connected each cell to its 10 nearest neighbors and were limited to cells of adjacent timepoints. Likewise, cell dissimilarity, which acts as repulsive forces between single cells within the FDL, was calculated based on angular (cosine) distance in indicated dimensions (fig. S2B).

Bulk RNA-seq analysis

Bulk RNA-seq_was performed by BGI America using the BGISEQ-500 platform, single end 50 bp-read length, at 30 x 10^6 reads per sample. Paired-end reads were aligned and quantified using Kallisto (version 0.44) (*72*) index against hg38 reference genome. The Gencode transcript annotations (version 27) were used for genomic location of transcriptomic units. Reads aligning to annotated regions were summarized as counts using R package tximport (version 1.12.3). Differential expression analyses of sample comparisons across various timepoints were performed using DESeq2 (version 1.24.0) (*73*). FDR cutoff of 0.05 was used for gene selection. Principal component analysis was performed on reads counts processed using variance-stabilizing transformation build into the DESeq2 package. PCA plots were generated using ggplot2 (2.3.2.1) in R (3.5.1). Gene set enrichment analysis was performed using the GSEA software (Broad Institute) as described (*74, 75*). Raw RNA-seq FASTQ files were used as input to perform quantitation of TCR receptor profiling using MiXCR tool (*76*). Briefly, reads were aligned to the V, D, J and C genes, assembled, and clonotypes were computed after filtering and error

correction steps. Clonotypes were further assessed using tcR, which enabled computation of clontype abundance and diversity estimations based on the CDR3 amino acid length and V gene usage (*77*). An outlier sample (one D15 Always OFF sample) sample was excluded from all RNA-seq analyses, as it did not cluster with replicates in a D15 PCA plot.

ATAC-seq data processing

ATAC-seq libraries were processed following the pepatac pipeline (http://code.databio.org/PEPATAC/) using default options and aligned to hg19. Briefly, fastq files were trimmed to remove Illumina Nextera adapter sequences, and pre-aligned to the mitochondrial genome to remove mitochondrial reads. Multi-mapping reads aligning to repetitive regions of the genome were also filtered. Bowtie2 was used to align to the hg19 genome. Samtools was used to identify uniquely aligned reads and Picard was used to remove duplicate reads. The resulting deduplicated aligned BAM file was used for downstream analysis. Peaks were called on individual samples by the pepatac pipeline using MACS2. A merged peak list was created . All individually-called peaks were compiled, then rank-ordered by signal intensity. Starting with the first peak, any overlapping peaks are removed; any remaining peaks then overlapping with the second-strongest peak are removed, and so on, until a set of unique non-overlapping peaks remained. These peaks were resized to a standard 500 bp width around the peak summit. Read counts in the merged peak set for each sample were generated with the bedtools intersect -c command. Merged signal tracks were made using aligned BAM files as inputs with the UCSC bigWigMerge and bedGraphToBigWig tools. The outlier samples (one D7 Always ON sample and one D15 Always OFF sample) were excluded from all ATAC-seq

analyses, including merged conditions, as they did not cluster with replicates (see below ATAC-seq analysis).

ATAC-seq analysis

The peak count table was pre-processed in R. It was depth normalized to account for differences in sequencing depth, then log-transformed with a pseudocount of 1. The resulting matrix was then quantile normalized across samples using the normalize.quantiles function in the preprocessCore library. Batch effects by patient were corrected with the removeBatchEffect function in the limma library. Differential peak calls across different conditions, using samples from different patients as replicates, were made using edgeR. P-values were adjusted by the Benjamini-Hochberg method. The number of significantly differentially accessible peaks was shown using the adjusted p-value less than or equal to 0.05 as a cutoff, aggregated by their logFC from the edgeR output (Fig. 5B and fig. S6, B and C).

Samples were initially analyzed based on their pairwise Pearson correlation to determine if replicates clustered as expected. Only peaks with a read in at least half (18/36) of the samples were included, and the correlation was computed on the log-transformed counts. One outlier each from the D7 Always ON and D15 Always OFF samples were observed and subsequently excluded from downstream analyses (Fig. S6D).

PCA plots were generated using the decomposition. PCA function in the sci-kit learn package in Python. PCA plots were generated using batch-corrected, quantile-normalized,

log-transformed peak counts (with a pseudocount of 1). Only peaks with at least 5 counts-per-million in at least 9/36 of the samples were used (Fig. 5D and fig. S6E).

Homer was used to compute relative motif enrichment of the peaks contributing to the loadings of the first three components of the PCA analysis described above. The 5000 peaks most contributing to the PC in either direction based on their loadings were used. These peaks represent the peaks whose accessibility across samples most contributes to a given principal component. The findMotifsGenome function in Homer was used using these peaks relative to the background of the merged peak set (not the entire genome, but the merged peak set generated as described above). The motif enrichment thus represents the specific enrichment of those PCs against the background of CAR-T cell specific peaks, not just CAR-T cell specific peaks enriched over the entire genome (Fig. 5E and fig. S6F).

K-means clustering was used to identify groups of peaks with similar accessibility dynamics. K-means clustering was performed on the D15 samples using only peaks that were significantly differentially accessible between any pair of D15 samples. The cluster.KMeans function in sci-kit learn was used with the following arguments: n_cluster=6, n_init=100, max_iter=100, precomputer_distances=True. 6 clusters were chosen based on k=6 representing the inflection point of the sum of squared errors decline as k was increased from 1 to 25. The peak accessibility data was z-normalized across samples prior to K-means clustering (Fig. S6G).

The GREAT (Genomic Regions Enrichment of Annotations Tool) v 3.0.0 was used to

infer putative biological functions from these sets of peaks (*78*), using their online tool (http://bejerano.stanford.edu/great/public/html/). The D15 differentially accessible peak set described in the previous paragraph was used was used as the background set, as opposed to the entire genome. Briefly, GREAT associates *cis*-regulatory regions with genes by first establishing a regulatory domain for any given gene, and then linking *cis*-regulatory elements with genes whose regulatory domains overlap. A foreground/background hypergeometric test is used to calculate the enrichment of the genes associated with a given peak set and annotation over the genes associated with the background peak set and annotation, using a variety of annotation ontologies included within GREAT, such as GO, PANTHER, and MSigDB.

Heatmaps are shown clustered using ward clustering and Euclidean distance. ChromVAR was used to compute the relative differential accessibility of known transcription factor motifs across different samples (*79*). Briefly, peaks counts were GC-corrected using the addGCBias function and the Jaspar motifs list was used to compute motif deviations with the getJasparMotifs, matchMotifs, and computeDeviations functions. Peak deviations were ranked by their variability across samples (standard deviation), and the most-variable deviations were shown. Note that some motifs are closely related and therefore behave similarly, and these closely related motifs were not collapsed or removed (Fig. 5E).

ChIP-seq analyses

Sequencing adapters were trimmed using fastp (*80*). Reads were aligned to the hg38 reference genome using hisat2 with the --no-spliced-alignment and --very-sensitive options

(*81*). PCR duplicates were removed in two steps. First, duplicates were removed from the original fastq files using the clumpify command line utility (part of the BBTools suite of bioinformatic tools) to decrease fastq file size before alignment. Then, duplicates after alignment were marked and removed by Picard. Peaks were called using MACS2 from the paired end bed file (bedpe) using a false discovery threshold of 0.05 (-q 0.05). A union peak set was created using our previous described workflow (*82*). Briefly, overlapping peaks were iteratively removed by removing less significant peaks until a set of disjoint, high confidence peaks across all samples was obtained. Normalized bigwig files were created by normalizing the coverage track of the bedpe file by reads in peaks. A peak by sample matrix was created by counting overlaps between reads and peaks for each sample and the resulting counts matrix was used as input into DESeq2 for differential peak analyses (*73*).

Statistical Analysis

Unless otherwise stated, statistical analyses for significant differences between groups were conducted using one- or two-way ANOVA and Dunnett's multiple comparisons test, or student's t test using GraphPad Prism 7. For all one-way ANOVA and student's t test analyses, we confirmed normality and equality of variance assumptions using Kolmogorov-Smirnoff and Shapiro-Wilk tests. Where these assumptions were not met, we replaced one-way ANOVA and student's t test with far more stringent and non-parametric tests, the Kruskal-Wallis test and Dunn's multiple comparisons test, and the Mann-Whitney test, respectively, to establish significance of difference between groups. In experiments where the same donor samples were being compared across different conditions, repeated

measures ANOVA/Friedman test or paired student's t test/Wilcoxon matched-pairs signed-rank test were performed. Experiments where data was measured at zero or below the limit of detection were excluded from normality assessment. Survival curves were compared using the Log-rank Mantel-Cox test.

Figure S1: GD2-targeting CARs fused to ecDHFR or FKBP destabilizing domains exhibit rapid ON/OFF kinetics and drug-dependent control of expression and function in vitro and in vivo. A) GD2.28ζ.ecDHFR CAR-T cells were stained with 1A7 anti-CAR idiotype antibody and analyzed via flow cytometry. Dose-dependent control (left) and ON/OFF kinetics (middle, right) were demonstrated via trimethoprim (TMP). CAR median fluorescent intensity (MFI) was used to generate non-linear dose-response curves. Error bars represent mean ± SEM of 3 individual donors. **B)** $1x10^6$ Nalm6-GD2 leukemia cells were engrafted in mice. On days 5-7 post-engraftment, mice were dosed with vehicle (water, CAR OFF) or 200mg/kg trimethoprim (TMP, CAR ON) once daily. On day 7 post-engraftment, $1x10^7$ GD2.28ζ.ecDHFR CAR-T cells were infused. 24 hours after CAR-T cell infusion, peripheral blood and spleens were harvested for flow cytometry analyses. Representative mouse from n=7 total mice from 2 independent experiments. **C)** Western blot showing total and phosphorylated CAR CD3ζ. Representative donor from n=3 individual donors. **D)** D11 flow cytometry showing inhibitory receptor expression **E)** Incucyte assay in which 143B osteosarcoma was mixed with GD2.28ζ.FKBP CAR-T cells (1:8 E:T, normalized to t=0). **(F)** IL-2 and IFNγ secretion from GD2.28ζ.FKBP CAR-T cells co-cultured with Nalm6-GD2 leukemia. Data represent the mean of triplicate wells (E-F) or the mean ± SD. Representative donor from 3 or more donors.

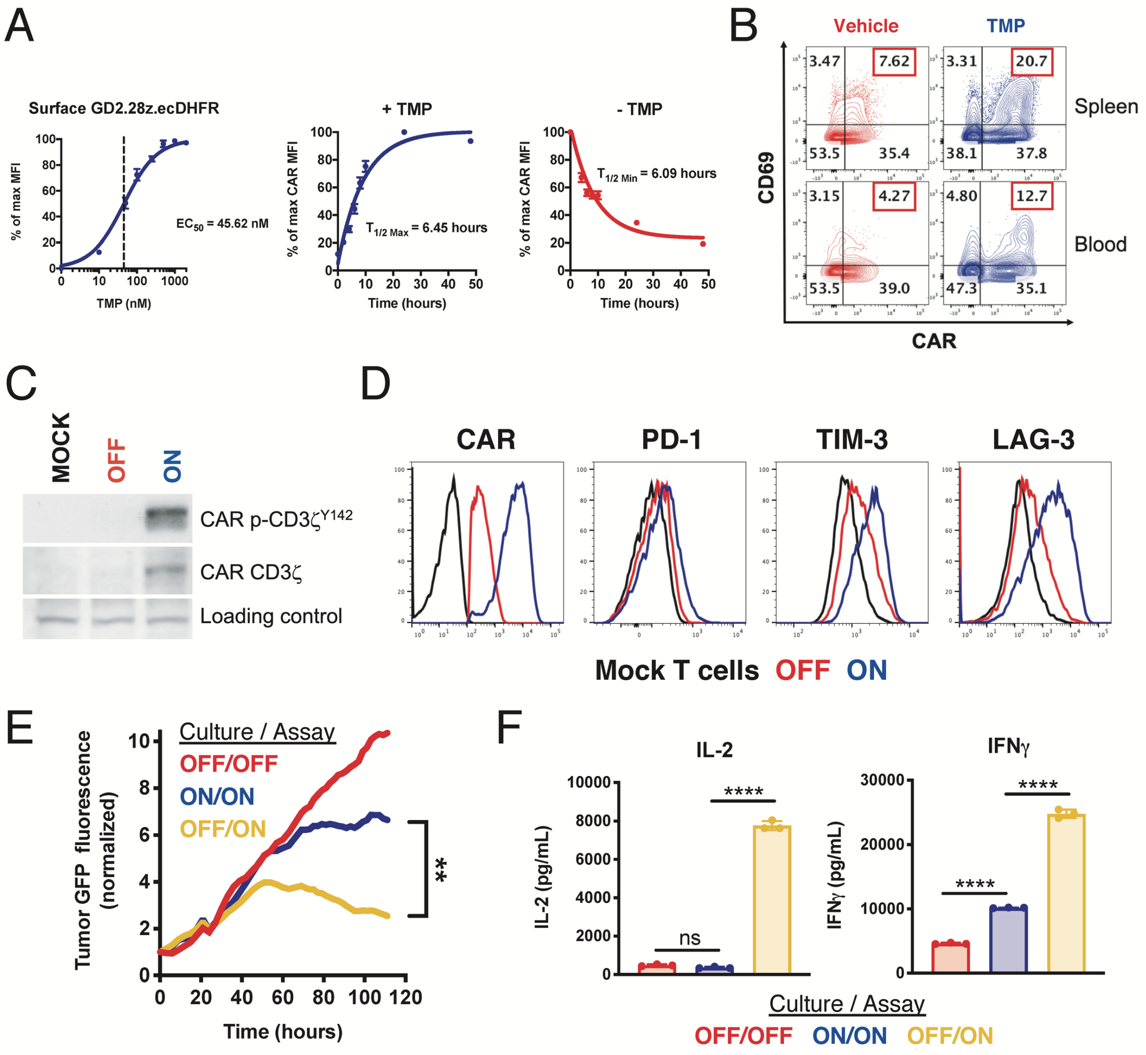

A
Surface GD2.28z.ecDHFR
EC50 = 45.62 nM
TMP (nM)
% of max MFI
+ TMP
T1/2 Max = 6.45 hours
- TMP
T1/2 Min = 6.09 hours
% of max CAR MFI
Time (hours)
B
Vehicle
TMP
Spleen
Blood
CD69
CAR
3.47
7.62
53.5
35.4
3.31
20.7
38.1
37.8
3.15
4.27
53.5
39.0
4.80
12.7
47.3
35.1
C
MOCK
OFF
ON
CAR p-CD3ζY142
CAR CD3ζ
Loading control
D
CAR
PD-1
TIM-3
LAG-3
Mock T cells OFF ON
E
Culture / Assay
OFF/OFF
ON/ON
OFF/ON
Tumor GFP fluorescence (normalized)
Time (hours)
**
F
IL-2
IFNγ
IL-2 (pg/mL)
IFNγ (pg/mL)
ns
****
Culture / Assay
OFF/OFF ON/ON OFF/ON

Figure S2: Mass cytometry and RNA-sequencing demonstrate population-wide phenotypic changes in rested CD8+ HA.28ζ.FKBP CAR-T cells. A) Flow cytometry of HA.28ζ CAR fused to an FKBP DD (HA.28ζ.FKBP) exhibits drug-dependent regulation of surface expression and rapid ON ($T_{1/2\ Max}$=6.09hr) and OFF kinetics ($T_{1/2\ Min}$=0.91hr). Error bars represent the mean ± SEM of 3 individual donors. **B)** Heavy metal-conjugated antibody panel used in mass cytometry experiments (27 antibodies, blue indicates markers used to generate the force directed layouts). **C)** Regions 1-4 (blue boxes) were identified based on the location of cells within the FDL on D11 (black box). **D)** CD39 expression over time in CD8+/CAR+ T cells. **E)** Heatmap demonstrates differences in cell phenotype between regions 1-4. Marker expression was normalized to the maximum value within each row. **F)** T-bet and Blimp-1 expression levels and co-expression frequency. **G)** Fold change in CAR-T cell expansion between D7 and D11. Statistics were calculated using paired two-tailed student's t test. **, p<0.01**. H)** Cleaved PARP-1 (cPARP) and Ki-67. Violin plots of single cells show quartiles with a band at the mean. **I)** D11 TCF1 expression via intracellular flow cytometry. TCF1+ gating was determined based on the TCF1+ naïve cell population. The histogram (top) shows data from 1 representative donor and the bar graph (bottom) represents the mean ± SEM of n=3 individual donors. **J-K)** MiXCR analyses of TCR clonality on D11 RNA-sequencing samples show **(J)** the relative abundance of clones for a representative donor and **(K)** Gini-Simpson Index measuring clonotypic diversity for 3 donors. FDLs and other measurements from mass cytometry experiments were derived from 1 representative donor (n=3 individual donors). Statistics were calculated via paired two-way student's t test (G and K), one-way ANOVA with

Tukey’s multiple comparisons test (I), or Wilcoxon matched-pairs signed-rank (K). **, $p<0.01$; ns, $p>0.05$.

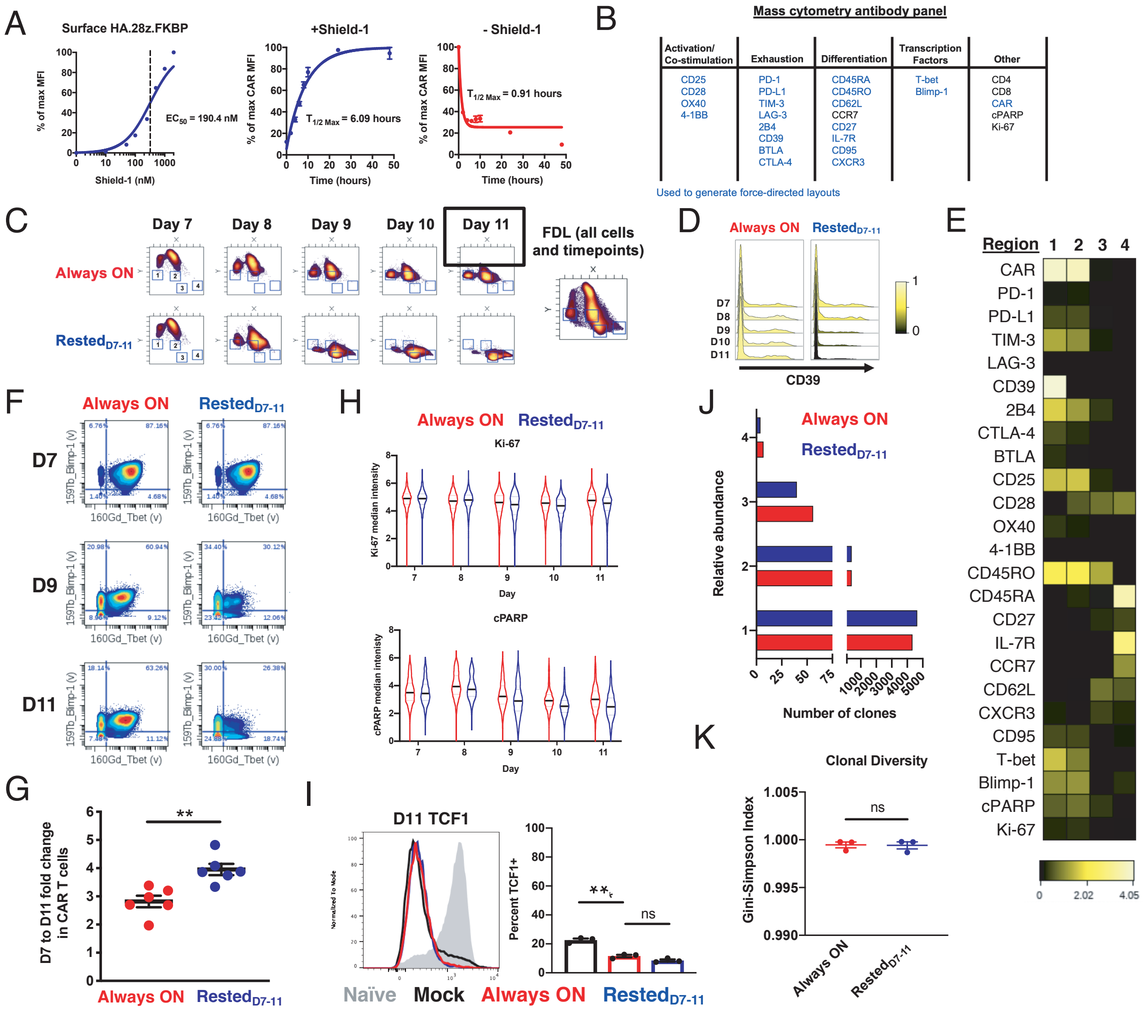

A
Surface HA.28z.FKBP
EC50 = 190.4 nM
Shield-1 (nM)
% of max MFI
+Shield-1
T1/2 Max = 6.09 hours
Time (hours)
% of max CAR MFI
- Shield-1
T1/2 Max = 0.91 hours
B
Mass cytometry antibody panel
Activation/ Co-stimulation
CD25
CD28
OX40
4-1BB
Exhaustion
PD-1
PD-L1
TIM-3
LAG-3
2B4
CD39
BTLA
CTLA-4
Differentiation
CD45RA
CD45RO
CD62L
CCR7
CD27
IL-7R
CD95
CXCR3
Transcription Factors
T-bet
Blimp-1
Other
CD4
CD8
CAR
cPARP
Ki-67
Used to generate force-directed layouts
C
Day 7
Day 8
Day 9
Day 10
Day 11
FDL (all cells and timepoints)
Always ON
RestedD7-11
D
CD39
E
Region
CAR
PD-1
PD-L1
TIM-3
LAG-3
CD39
2B4
CTLA-4
BTLA
CD25
CD28
OX40
4-1BB
CD45RO
CD45RA
CD27
IL-7R
CCR7
CD62L
CXCR3
CD95
T-bet
Blimp-1
cPARP
Ki-67
F
D7
D9
D11
160Gd_Tbet (v)
159Tb_Blimp-1 (v)
H
Ki-67
Ki-67 median intensity
cPARP
cPARP median intensity
Day
J
Relative abundance
Number of clones
K
Clonal Diversity
Gini-Simpson Index
ns
G
D7 to D11 fold change in CAR T cells
**
I
D11 TCF1
Percent TCF1+
Naïve
Mock
Always ON
RestedD7-11

Figure S3: Flow and mass cytometry of rested CD4+ HA.28ζ.FKBP CAR-T cells. A) Inhibitory receptor expression of CD4+ HA.28ζ.FKBP CAR-T cells via flow cytometry. Error bars represent mean ± SEM of 3 individual donors. **B-E)** Force-directed layouts (FDL) were constructed from 20,000 CAR+/CD4+ events analyzed by mass cytometry using the panel shown in fig. S2B. Events were equally sampled from Always ON and $Rested_{D7-11}$ cells collected on each day between D7-11 (2,000 cells per condition/timepoint). Cells repel based on expression of 20 surface and 2 intracellular markers (Figure S2B) and grey edges connect cells from adjacent days (1 representative donor from 3 individual donors). **B)** Regions 1-3 (blue boxes) were identified based on the location of cells within the FDL on D11. **C)** Timepoint analyses demonstrate that D11 cells are concentrated in phenotypically distinct regions 1-3, whereby regions 1 and 2 primarily consist of D11 Always ON cells (95% and 93%, respectively), while region 3 contains almost entirely of D11 $Rested_{D7-11}$ cells (95%). **D)** FDLs overlaid with expression of exhaustion/activation markers CD39, CD25, and Blimp-1. **E)** FDLs overlaid with expression of memory markers CD45RA, CD62L, and CXCR3. Statistics were calculated using one-way ANOVA and Dunnett's multiple comparisons test (C and F, left) or Kruskal-Wallis and Dunn's multiple comparisons test (F, right) or Friedman test and Dunn's multiple comparisons test (L). *, $p<0.05$; **, $p<0.01$; ***, $p<0.001$; ****, $p<0.0001$; ns, $p>0.05$

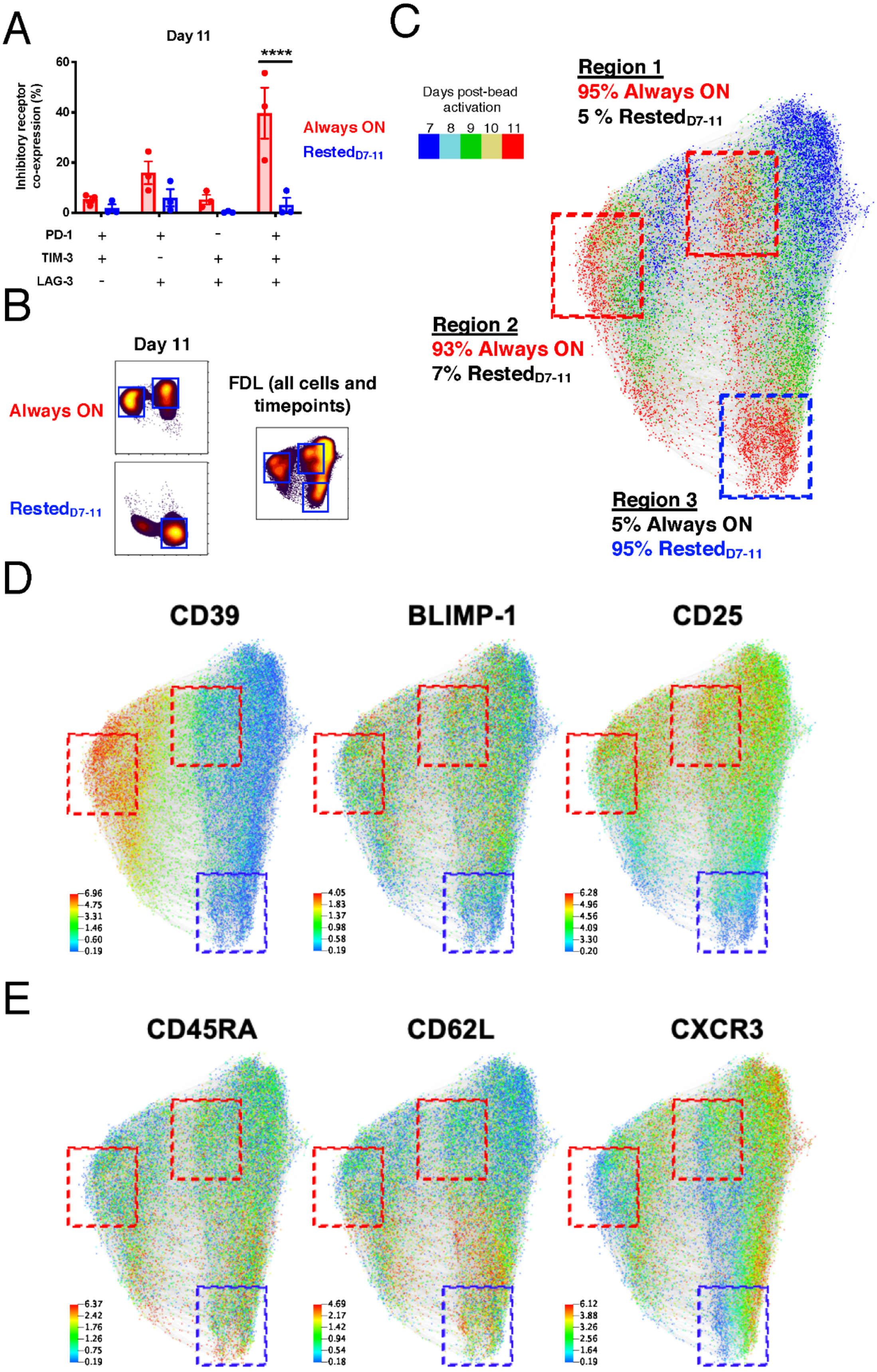

A
Day 11
Inhibitory receptor co-expression (%)
60
40
20
0
****
Always ON
Rested$_{D7-11}$
PD-1 + + - +
TIM-3 + - + +
LAG-3 - + + +
B
Day 11
Always ON
Rested$_{D7-11}$
FDL (all cells and timepoints)
C
Days post-bead activation
7 8 9 10 11
Region 1
95% Always ON
5 % Rested$_{D7-11}$
Region 2
93% Always ON
7% Rested$_{D7-11}$
Region 3
5% Always ON
95% Rested$_{D7-11}$
D
CD39
BLIMP-1
CD25
6.96 4.75 3.31 1.46 0.60 0.19
4.05 1.83 1.37 0.98 0.58 0.19
6.28 4.96 4.56 4.09 3.30 0.20
E
CD45RA
CD62L
CXCR3
6.37 2.42 1.76 1.26 0.75 0.19
4.69 2.17 1.42 0.94 0.54 0.18
6.12 3.88 3.26 2.56 1.64 0.19

Figure S4: Phenotypic and transcriptional reprogramming of exhausted CAR-T cells via rest. A) HA.28ζ.FKBP CAR-T cells collected on D15 were lysed for western blot analysis of total and phosphorylated CAR CD3ζ and ERK1/2. Representative donor from 3 individual donors. **B)** CAR-T cells were loaded with cell trace violet (CTV) on D11 and analyzed on D15 (representative donor of n=3 individual donors). **C-D)** Flow cytometry demonstrates reversal of inhibitory receptor expression to Always OFF levels upon rest, regardless of whether rest is started at D7 ($Rested_{D7\text{-}11}$, $Rested_{D7\text{-}15}$) or D11 ($Rested_{D11\text{-}15}$). (C) displays mean ± SEM of 6 individual donors and (D) displays one representative donor from 3 individual donors. **E-F)** CD45RO and CCR7 expression on CD4+ CAR+ T cells. (E) Flow cytometry contour plots are representative of 3 individual donors and (F) bar graphs show mean ± SEM of 3 individual donors. **G-J)** Bulk RNA sequencing data of unsorted HA.28ζ.FKBP CAR-T cells (Figures 4E-G). **G)** Data analysis pipeline for generating an Always ON exhaustion signature, which is comprised of 708 and 442 transcripts that are up- or downregulated in Always ON T cells, respectively. **H)** Principal component analyses of D7 (left) and D11 (right) RNA-sequencing data. **I)** Volcano plot of differentially expressed genes between $Rested_{D11\text{-}15}$ cells and Always ON cells on D15. Genes were filtered for those that exhibited log2(fold change)>0.5 or <-0.5 and p(adjusted)<0.05. **J)** GSEA: gene sets from Always OFF, $Rested_{D7\text{-}15}$ and $Rested_{D11\text{-}15}$ cells compared to genes sets from Exhausted or Memory CD8+ T cells (top) in a mouse model of chronic LCMV (*41*). GSEA – gene set enrichment analysis, NES – normalized enrichment score, FDR – false discovery rate. **K-L)** MiXCR analyses of TCR clonality on D15 RNA-seq samples show (K) the relative abundance of clones for each sample and (L)

Gini-Simpson Index measuring clonotypic diversity for 3 donors. Bracketed bar in (L) indicates the same statistical significance for all sample comparisons. **M)** Mass cytometry of cleaved PARP (cPARP) on D15 (n=3 individual donors). Statistics were calculated using one-way ANOVA and Dunnett's (C and F) or Tukey's (L) multiple comparison's test. *, $p<0.05$; **, $p<0.01$ ***, $p<0.001$; ****$p<0.0001$; ns, $p>0.05$

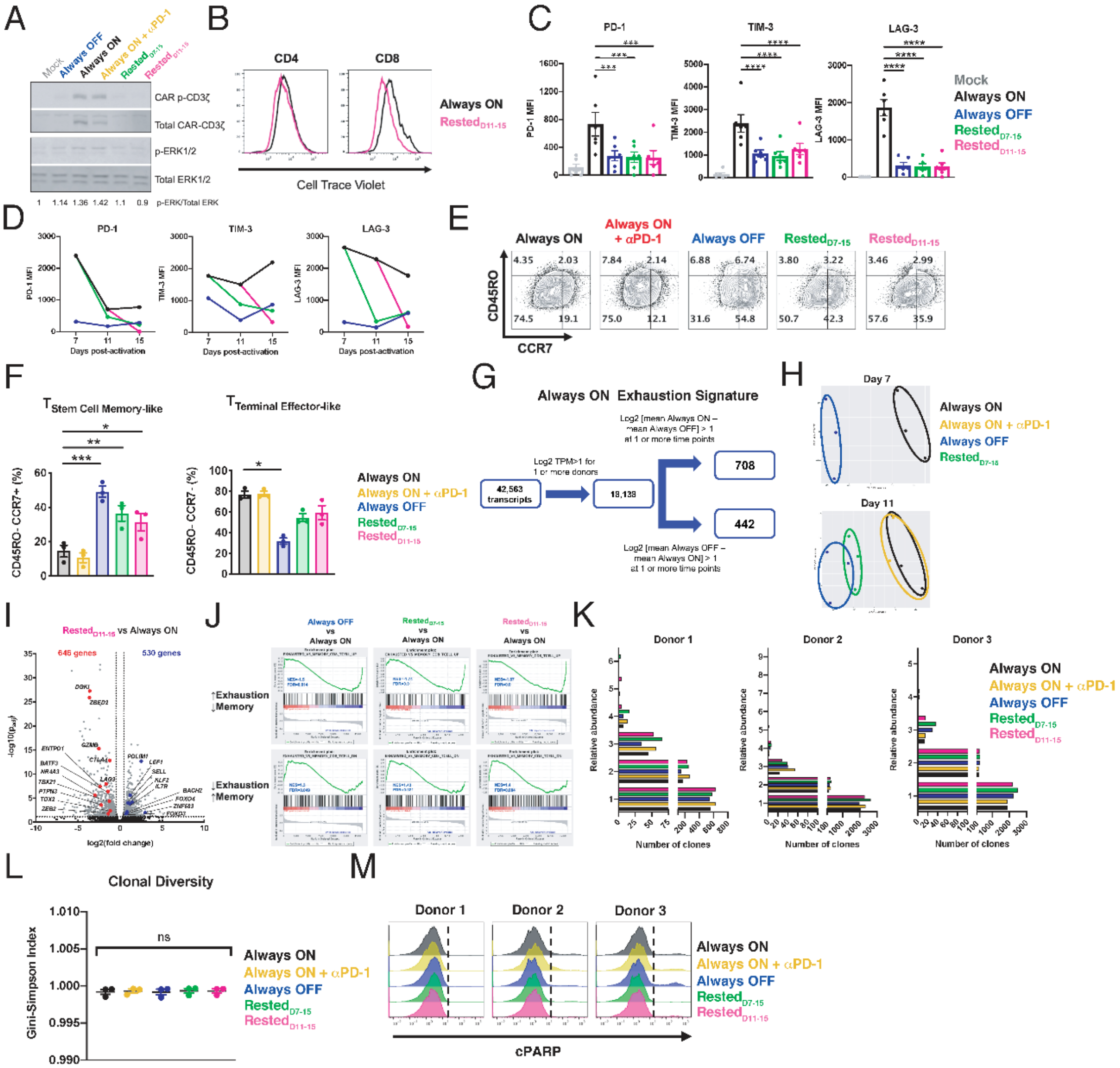
A
Mock
Always OFF
Always ON
Always ON + αPD-1
Rested D7-15
Rested D11-15
CAR p-CD3ζ
Total CAR-CD3ζ
p-ERK1/2
Total ERK1/2
1 1.14 1.36 1.42 1.1 0.9 p-ERK/Total ERK
B
CD4
CD8
Always ON
Rested D11-15
Cell Trace Violet
C
PD-1
TIM-3
LAG-3
PD-1 MFI
TIM-3 MFI
LAG-3 MFI
Mock
Always ON
Always OFF
Rested D7-15
Rested D11-15
D
PD-1
TIM-3
LAG-3
Days post-activation
E
Always ON
Always ON + αPD-1
Always OFF
Rested D7-15
Rested D11-15
CD45RO
CCR7
4.35 2.03 74.5 19.1
7.84 2.14 75.0 12.1
6.88 6.74 31.6 54.8
3.80 3.22 50.7 42.3
3.46 2.99 57.6 35.9
F
T Stem Cell Memory-like
T Terminal Effector-like
CD45RO- CCR7+ (%)
CD45RO- CCR7- (%)
Always ON
Always ON + αPD-1
Always OFF
Rested D7-15
Rested D11-15
G
Always ON Exhaustion Signature
42,563 transcripts
Log2 TPM>1 for 1 or more donors
18,138
Log2 [mean Always ON – mean Always OFF] > 1 at 1 or more time points
708
442
Log2 [mean Always OFF – mean Always ON] > 1 at 1 or more time points
H
Day 7
Day 11
Always ON
Always ON + αPD-1
Always OFF
Rested D7-15
I
Rested D11-15 vs Always ON
646 genes
530 genes
log2(fold change)
J
Always OFF vs Always ON
Rested D7-15 vs Always ON
Rested D11-15 vs Always ON
↑Exhaustion ↓Memory
↓Exhaustion ↑Memory
K
Donor 1
Donor 2
Donor 3
Relative abundance
Number of clones
Always ON
Always ON + αPD-1
Always OFF
Rested D7-15
Rested D11-15
L
Clonal Diversity
Gini-Simpson Index
ns
Always ON
Always ON + αPD-1
Always OFF
Rested D7-15
Rested D11-15
M
Donor 1
Donor 2
Donor 3
cPARP
Always ON
Always ON + αPD-1
Always OFF
Rested D7-15
Rested D11-15

Figure S5: Transient rest restores functionality in exhausted CAR-T cells. A) Human naïve T cells were FACS sorted ($CD45RA^{+}CD45RO^{-}$, $CD62L^{+}$, $CCR7^{+}$, $CD95^{-}$, and $CD122^{-}$), transduced to express GD2.28ζ.FKBP, and cultured with or without shield-1, or rested for 3-7 days, as illustrated in Figure 3A. **B-C)** Intracellular cytokine staining and flow cytometry analyses of CD4+ and CD8+ HA.28ζ.FKBP CAR-T cells activated with 143B-GL demonstrate (B) the frequency of CD4+ non-responsive and polyfunctional cytokine-secreting cells, and (C) the frequency of CD4+ and CD8+ IL-2, IFNγ, or TNFα positive cells. SPICE analysis from 1 representative donor was conducted in (B). Error bars in (B) and (C) represent mean ± SEM of 3 individual donors. Representative histograms in (C) are shown. **D)** Dose-dependent control of HA.28ζ.ecDHFR expression (left) and ON/OFF kinetics (middle, right) were demonstrated via trimethoprim (TMP) treatment. CAR MFI was used to generate non-linear dose-response curves. Error bars represent mean ± SEM of 2-3 individual donors. **E)** Flow cytometry of HA.28ζ.ecDHFR conditioned as described in Fig. 4A. **F)** Quantification of tumor bioluminescence data from the experiments described in Figures 4H and 4I. Representative experiment from 3 individual experiments (n=3-5 mice/group). Statistics were calculated using one- or two-way ANOVA and Dunnett's multiple comparisons test. *, $p<0.05$; **, $p<0.01$ ***, $p<0.001$; ****$p<0.0001$

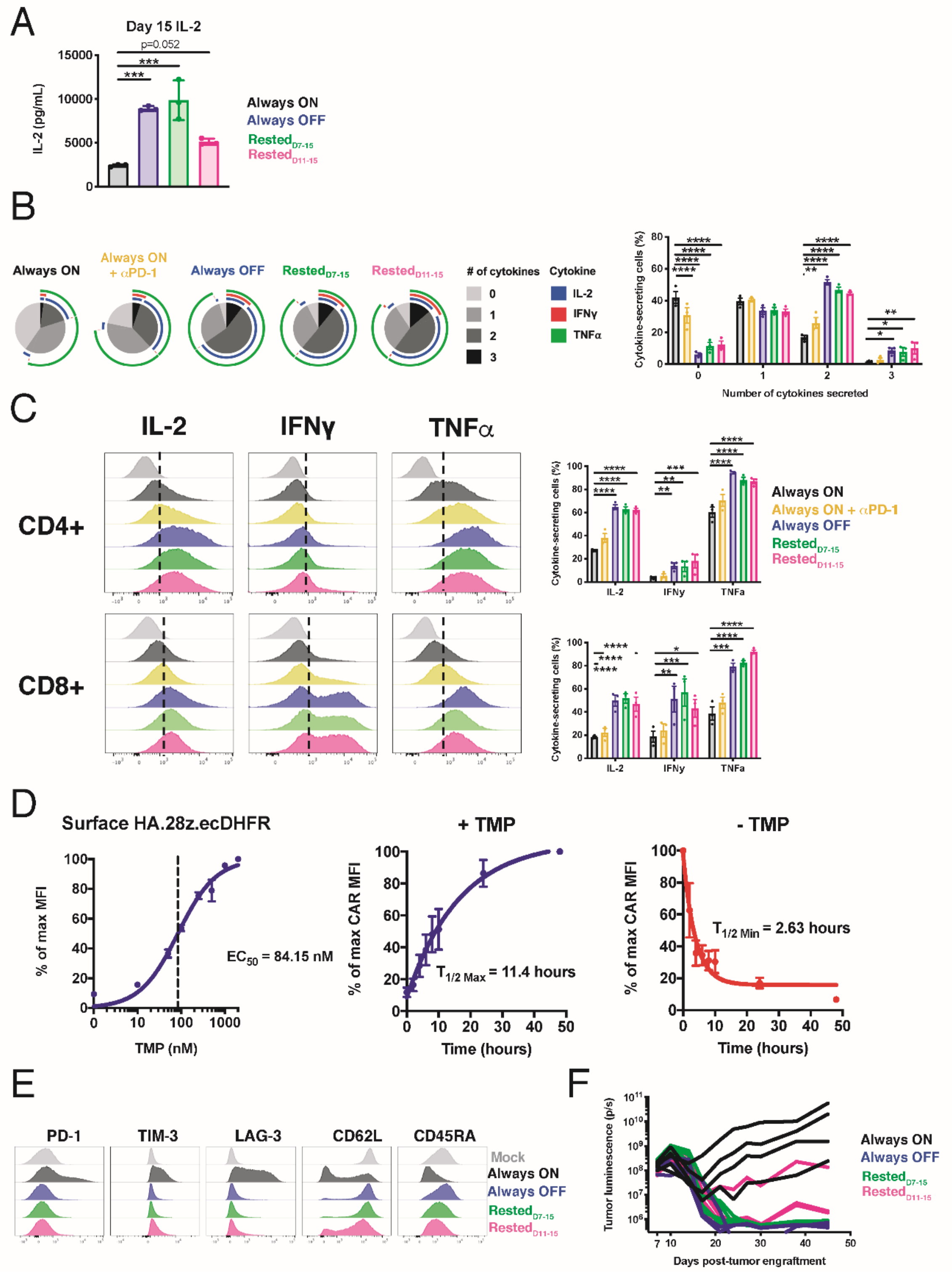
A
Day 15 IL-2
p=0.052
***
***
IL-2 (pg/mL)
Always ON
Always OFF
Rested D7-15
Rested D11-15
B
Always ON
Always ON + αPD-1
Always OFF
Rested D7-15
Rested D11-15
# of cytokines
Cytokine
IL-2
IFNγ
TNFα
Cytokine-secreting cells (%)
Number of cytokines secreted
C
IL-2
IFNγ
TNFα
CD4+
CD8+
Cytokine-secreting cells (%)
Always ON
Always ON + αPD-1
Always OFF
Rested D7-15
Rested D11-15
D
Surface HA.28z.ecDHFR
% of max MFI
EC50 = 84.15 nM
TMP (nM)
+ TMP
% of max CAR MFI
T1/2 Max = 11.4 hours
Time (hours)
- TMP
T1/2 Min = 2.63 hours
E
PD-1
TIM-3
LAG-3
CD62L
CD45RA
Mock
Always ON
Always OFF
Rested D7-15
Rested D11-15
F
Tumor luminescence (p/s)
Days post-tumor engraftment
Always ON
Always OFF
Rested D7-15
Rested D11-15

Figure S6: Epigenetic reprogramming of exhausted CAR-T cells via rest. A) Transcriptional start site (TSS) enrichment scores for all samples demonstrate good quality control for the ATAC-seq data set. **B-C)** (B) Unbiased principal component analyses and (C) differentially accessible regions of activation-associated (*TNFRSF9*, *IL-2*) or exhaustion-associated (*JUNB*) genes are shown for D15 Always ON CAR-T cells and CD19.28ζ CAR-T cells that were unstimulated or crosslinked with immobilized anti-FMC63 idiotype antibody for 24-hours (stimulated). (C) shows 1 representative donor from n=3 individual donors. **D)** Peak accessibility changes between timepoints were calculated based on p adjusted<0.05. Bar graphs display merged data from 2-3 donors. **E)** D15 differentially accessible peaks compared to Always ON (FDR<0.05). **F)** Pearson correlation and hierarchical clustering of ATAC-sequencing data from each condition and timepoint outlined in Figure 6A. Heatmap generated with merged data from 2-3 individual donors. **G)** Unbiased principal component analysis of chromatin accessibility was assessed on individual time points D7, D11, and D15. Plots were generated with ATAC-seq data from 2-3 individual donors. **H)** TF motifs enriched in the top 5000 peaks that are negatively associated with PC1 in the principal component analysis in Figure 5D indicate a strong AP-1 family signature in exhausted Always ON and Always ON + αPD-1 samples. **I)** K-means clustering of D15 differentially accessible peaks identified 6 distinct clusters. GREAT (Genomic Regions Enrichment of Annotations Tool) was then used to assign genes to peaks, then infer putative biological functions from these sets of peaks/genes.

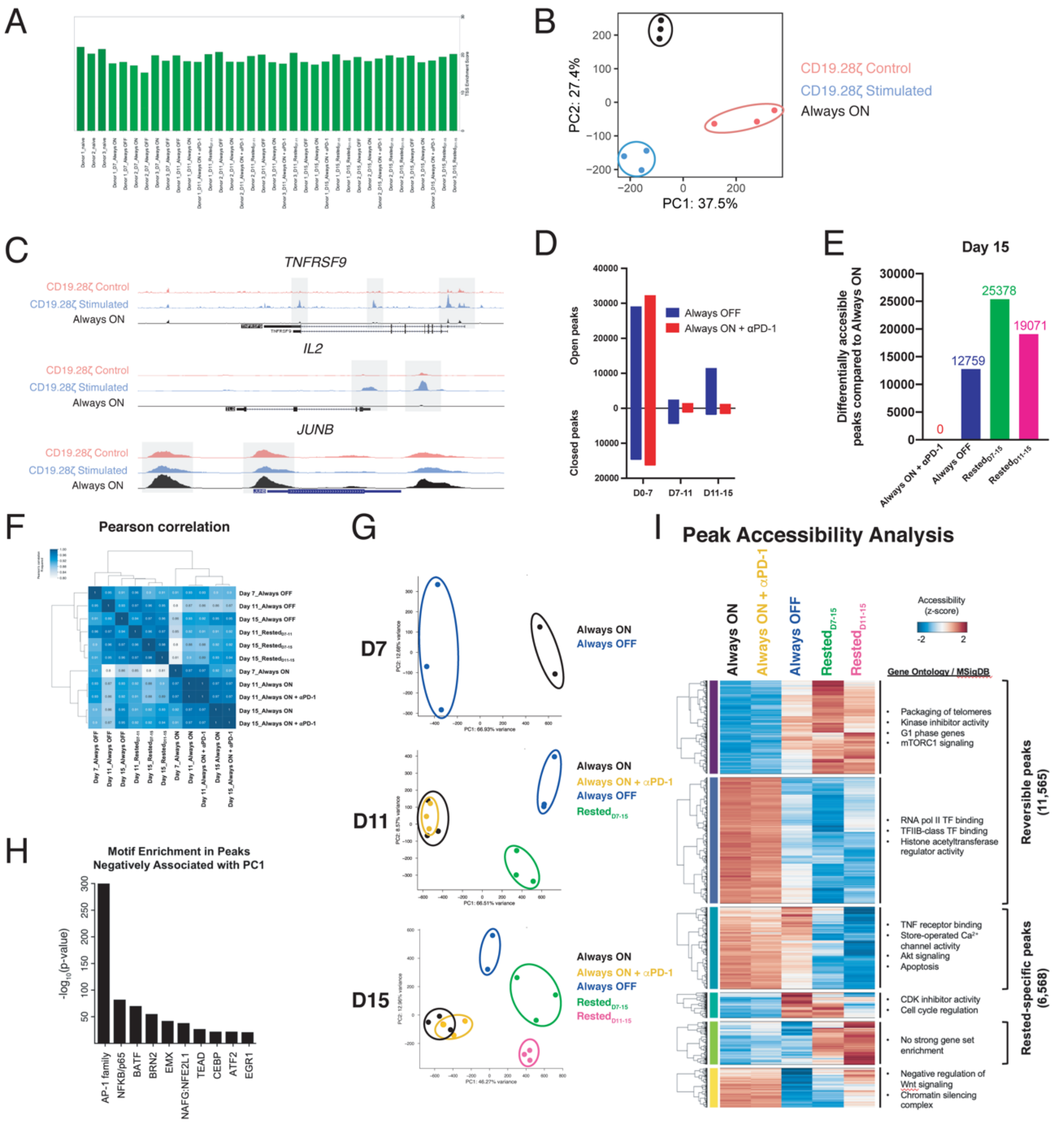
A
B
CD19.28ζ Control
CD19.28ζ Stimulated
Always ON
PC2: 27.4%
PC1: 37.5%
C
TNFRSF9
IL2
JUNB
D
Open peaks
Closed peaks
Always OFF
Always ON + αPD-1
D0-7
D7-11
D11-15
E
Day 15
Differentially accesible peaks compared to Always ON
0
12759
25378
19071
Always ON + αPD-1
Always OFF
Rested D7-15
Rested D11-15
F
Pearson correlation
G
D7
D11
D15
Always ON
Always ON + αPD-1
Always OFF
Rested D7-15
Rested D11-15
H
Motif Enrichment in Peaks Negatively Associated with PC1
-log10(p-value)
AP-1 family
NFKB/p65
BATF
BRN2
EMX
NAFG:NFE2L1
TEAD
CEBP
ATF2
EGR1
I
Peak Accessibility Analysis
Accessibility (z-score)
Gene Ontology / MSigDB
Packaging of telomeres
Kinase inhibitor activity
G1 phase genes
mTORC1 signaling
RNA pol II TF binding
TFIIB-class TF binding
Histone acetyltransferase regulator activity
TNF receptor binding
Store-operated Ca2+ channel activity
Akt signaling
Apoptosis
CDK inhibitor activity
Cell cycle regulation
No strong gene set enrichment
Negative regulation of Wnt signaling
Chromatin silencing complex
Reversible peaks (11,565)
Rested-specific peaks (6,568)

Figure S7: Differential effects of EZH2$_i$ on Rested$_{D11-15}$ CAR-T cells. Always ON, Always OFF, and Rested$_{D11-15}$ CAR-T cells were cultured with 0.1-1 μM or 1 μM (A through I) tazemetostat (EZH2$_i$), 1 μM decitabine (DNA methyltransferase inhibitor, C) 1 μM entinostat (histone deacetylase inhibitor), or vehicle from D11-15. Shield-1 was added to CAR-T cells approximately 16 hours prior to co-culture with tumor, and EZH2$_i$/tazemetostat, decitabine, and entinostat were washed out immediately prior to co-culture. **A)** Western blot analysis demonstrates a global reduction in H3K27me3 in EZH2$_i$-treated CAR-T cells (representative donor from n=2 individual donors). **B)** Chromatin immunoprecipitation sequencing (ChIP-seq) tracks exhibit reduced H3K27me3 peaks within exhaustion-associated loci (n=3 individual donors). **C)** Always OFF and Rested$_{D11-15}$ IL-2 secretion in response to tumor challenge. Data show mean ± SEM of 2 individual donors each challenged against both Nalm6-GD2 and 143B (n=4). **D)** D15 IFNγ secretion in response to Nalm6-GD2. Data shows mean ± SD of triplicate wells from 1 representative donor (n=4 individual donors). **E)** D15 CAR-T cell viability. Data shows mean ± SEM of 3 individual donors. **F)** Fold change in CAR-T cell expansion from D11 to D15. Data shows mean ± SEM of 3 individual donors. **G-H)** (G) Antigen-independent CAR-T cell proliferation from D11 to D15 and (H) antigen-dependent proliferation in CAR-T cells stimulated with 143B on D15 and analyzed on D18. Cells were loaded with trace violet dye and analyzed 3-4 days later via FACS. Data shows histograms from 1 representative donor (n=3 donors). **I)** D15 FACS analyses of CD8+ Rested$_{D11-15}$ CAR-T cells treated

with increasing concentrations of demonstrate that $EZH2_i$ (histograms from 1 representative donor of n=4 individual donors). Statistics were calculated using two-way ANOVA and Bonferroni multiple comparison's test. ns, $p>0.05$

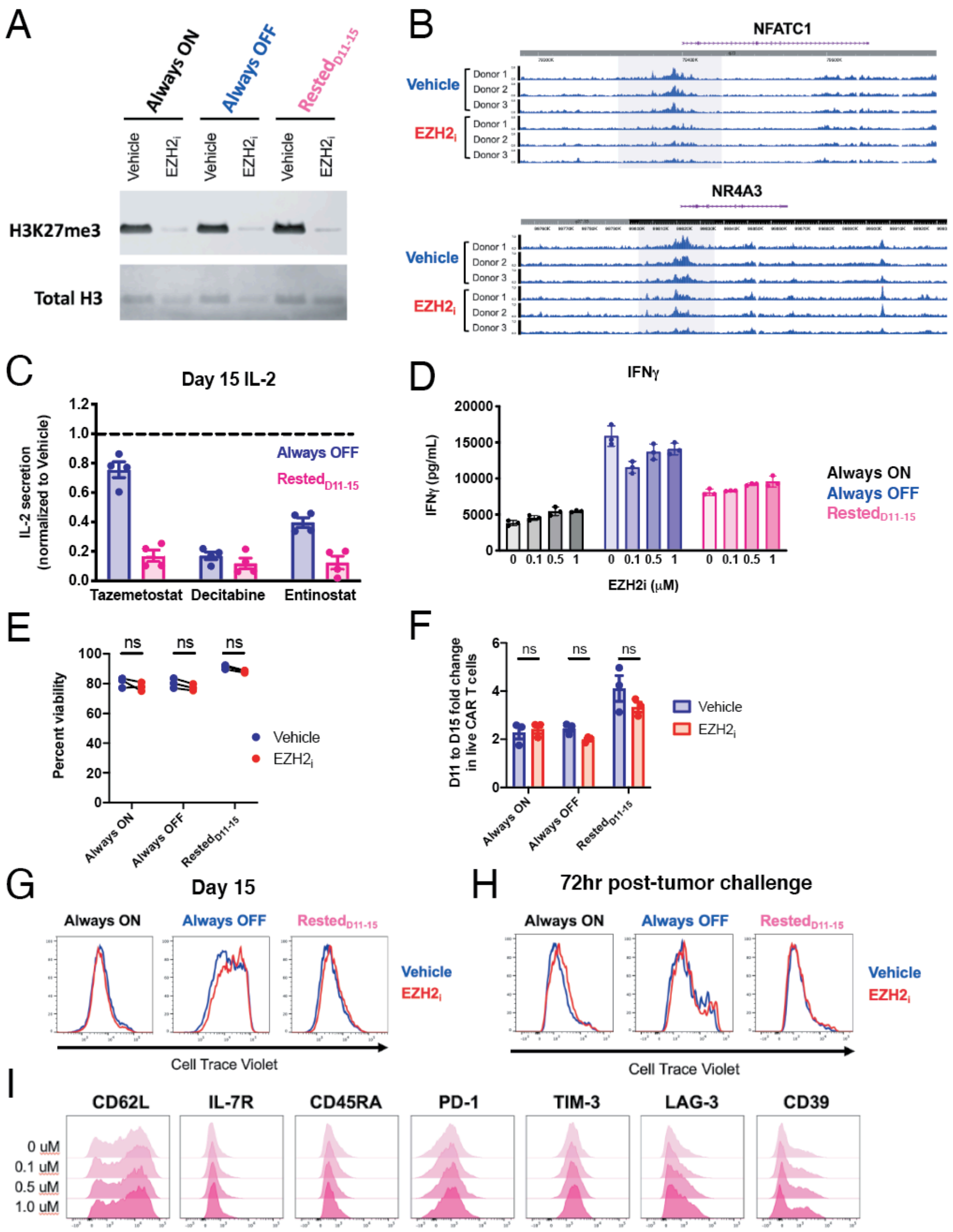
A
Always ON
Always OFF
Rested D11-15
Vehicle
EZH2i
H3K27me3
Total H3
B
NFATC1
Vehicle
EZH2i
Donor 1
Donor 2
Donor 3
NR4A3
C
Day 15 IL-2
IL-2 secretion (normalized to Vehicle)
Always OFF
Rested D11-15
Tazemetostat
Decitabine
Entinostat
D
IFNγ
IFNγ (pg/mL)
EZH2i (μM)
Always ON
Always OFF
Rested D11-15
E
Percent viability
ns
Vehicle
EZH2i
F
D11 to D15 fold change in live CAR T cells
G
Day 15
H
72hr post-tumor challenge
Cell Trace Violet
I
CD62L
IL-7R
CD45RA
PD-1
TIM-3
LAG-3
CD39
0 uM
0.1 uM
0.5 uM
1.0 uM

Figure S8: Dasatinib prevents tonic CAR signaling, reverses the exhaustion phenotype, and restores functionality in exhausted CAR-T cells. A) Western blot analysis of total and phosphorylated CD3ζ and ERK1/2 in D15 HA.28ζ CAR-T cells cultured in the presence of vehicle or dasatinib from D4-15 ($Dasatinib_{D4-15}$). Representative donor from n=3 individual donors. **B)** HA.28ζ CAR-T cell expansion from D0 to D15 in Vehicle and $Dasatinib_{D11-15}$ CAR-T cells (n=5 individual donors). **C)** Inhibitory receptor expression on D15 CD8+ CAR+ T cells via flow cytometry. Histograms show 1 representative donor of 3 individual donors. **D)** Dasatinib was removed from CAR-T cell cultures approximately 16 hours prior to co-culture with Nalm6-GD2 leukemia on D15, and IL-2 and IFNγ secretion was subsequently assessed. Error bars represent mean ± SD of triplicate wells from 1 representative donor (n=3 donors). **E)** Flow cytometry of memory markers in CD8+ GD2.BBζ CAR-T cells on D11 (representative donor of n=4 individual donors). **F)** $1x10^6$ 143B-GL osteosarcoma cells were engrafted in the flank of NSG mice, and $1x10^7$ CAR+ GD2.BBζ CAR-T cells that had been cultured in the presence of vehicle (DMSO) or dasatinib for 7 days were infused on day 3 post-engraftment. Tumor size was monitored via caliper measurements. Error bars represent mean ± SEM of 5 individual mice from 1 representative experiment from n=3 independent experiments. **G-H)** Quantification of D25 **(G)** exhaustion marker and **(H)** transcription factor expression via flow cytometry. Error bars represent mean MFI ± SEM of 3-4 individual donors. Bracketed horizontal bars indicate the same statistical significance across all conditions compared to Vehicle controls. **I)** IL-2 and IFNγ secretion before (D15) and after (D22) treatment with vehicle or dasatinib. Data represent 4 individual donors. **J)**

Co-culture assays with Nalm6-GD2 leukemia were conducted on D15, D18, D22, and D25. IL-2 and IFNγ secretion levels from CAR-T cells cultured with dasatinib for 7-8 days were normalized to $\text{Dasatinib}_{\text{D4-25}}$ for each individual timepoint. Graphs display data from 4-5 individual donors. These data are also represented in Figure 6G, but were replotted here to illustrate cytokine secretion when duration of rest is normalized across conditions. **K)** HA.28ζ.FKBP CAR-T cells were cultured in a protracted in vitro time course, as outlined in Fig. 6B. Error bars represent mean ± SD of 2-3 wells from one representative donor (n=3 individual donors). Statistics were calculated using paired student's t test (B) or one- or two-way ANOVA and Dunnett's (D, G, H, and K), Bonferroni's (I), or Tukey's multiple comparisons test (J). *, p<0.05; **, p<0.01 ***, p<0.001; ****p<0.0001; ns, p>0.05

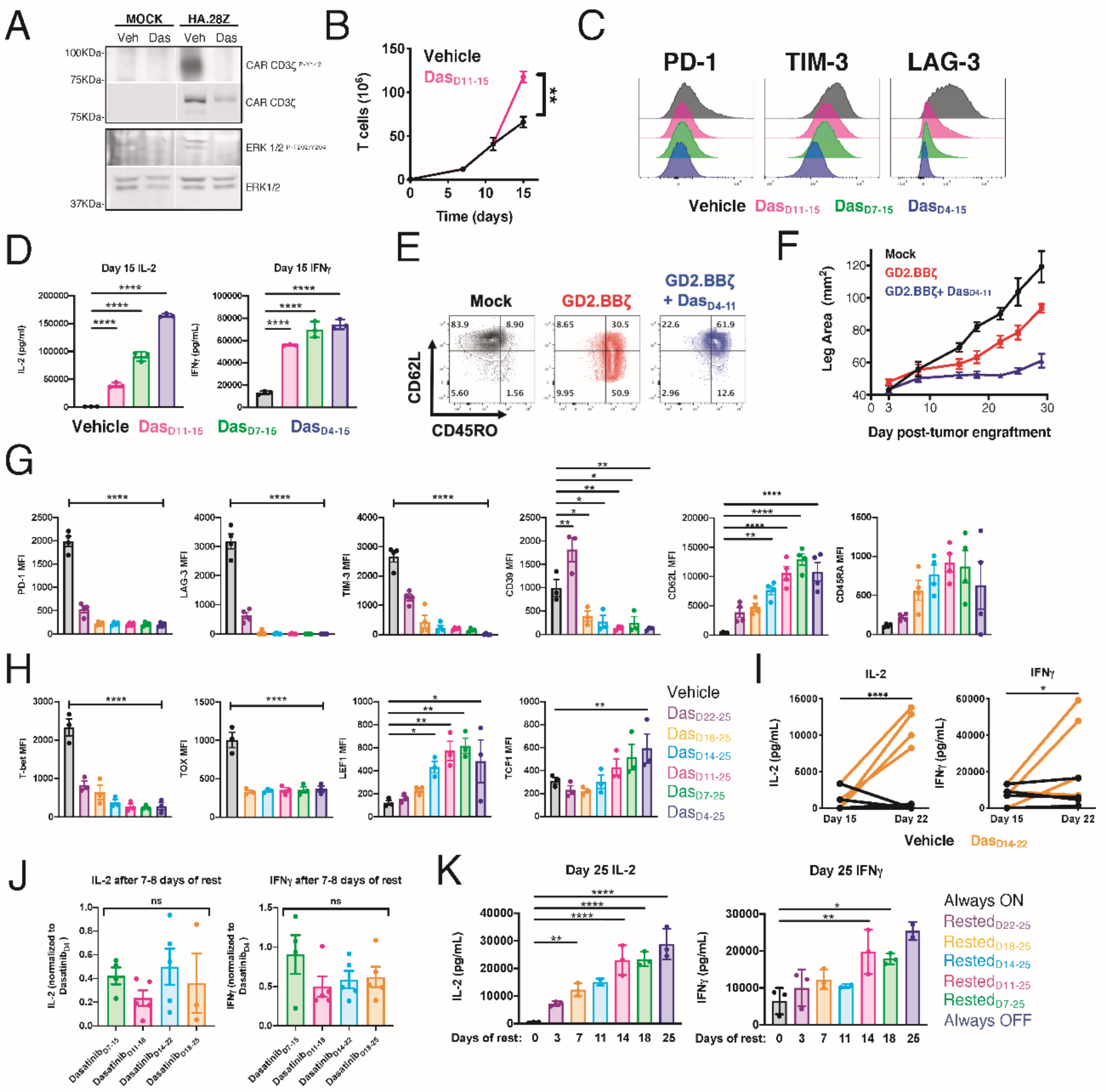

A
MOCK
HA.28Z
Veh
Das
CAR CD3ζ
ERK1/2
B
Vehicle
T cells (10^6)
Time (days)
C
PD-1
TIM-3
LAG-3
Vehicle
D
Day 15 IL-2
Day 15 IFNγ
E
Mock
GD2.BBζ
CD62L
CD45RO
F
Mock
GD2.BBζ
Leg Area (mm²)
Day post-tumor engraftment
G
H
I
IL-2
IFNγ
Day 15
Day 22
J
IL-2 after 7-8 days of rest
IFNγ after 7-8 days of rest
K
Day 25 IL-2
Day 25 IFNγ
Days of rest:
Always ON
Always OFF

Figure S9: Reinvigoration of very late time point exhausted CAR-T cells using dasatinib. A) Human CAR-T cells expressing HA.28ζ were cultured with vehicle (DMSO) or dasatinib for 7 or 24 days starting on day 46 or day 29, respectively. Cells were collected on day 53 for FACS phenotypic analyses. **B-E)** D53 flow cytometry showing (B) exhaustion marker expression (C) memory phenotype, and the (D) frequency and (E) magnitude of expression of exhaustion-associated transcription factors T-bet and TOX. Histograms and biaxial dot plots are 1 representative donor and error bars represent mean ± SEM of n=3 individual donors. Statistics were calculated using one-way ANOVA and Dunnett's multiple comparisons test (B, C, and E, bottom) or Friedman test and Dunn's multiple comparisons test (E, top). *, $p<0.05$; **, $p<0.01$; ***, $p<0.001$; ns, $p>0.05$

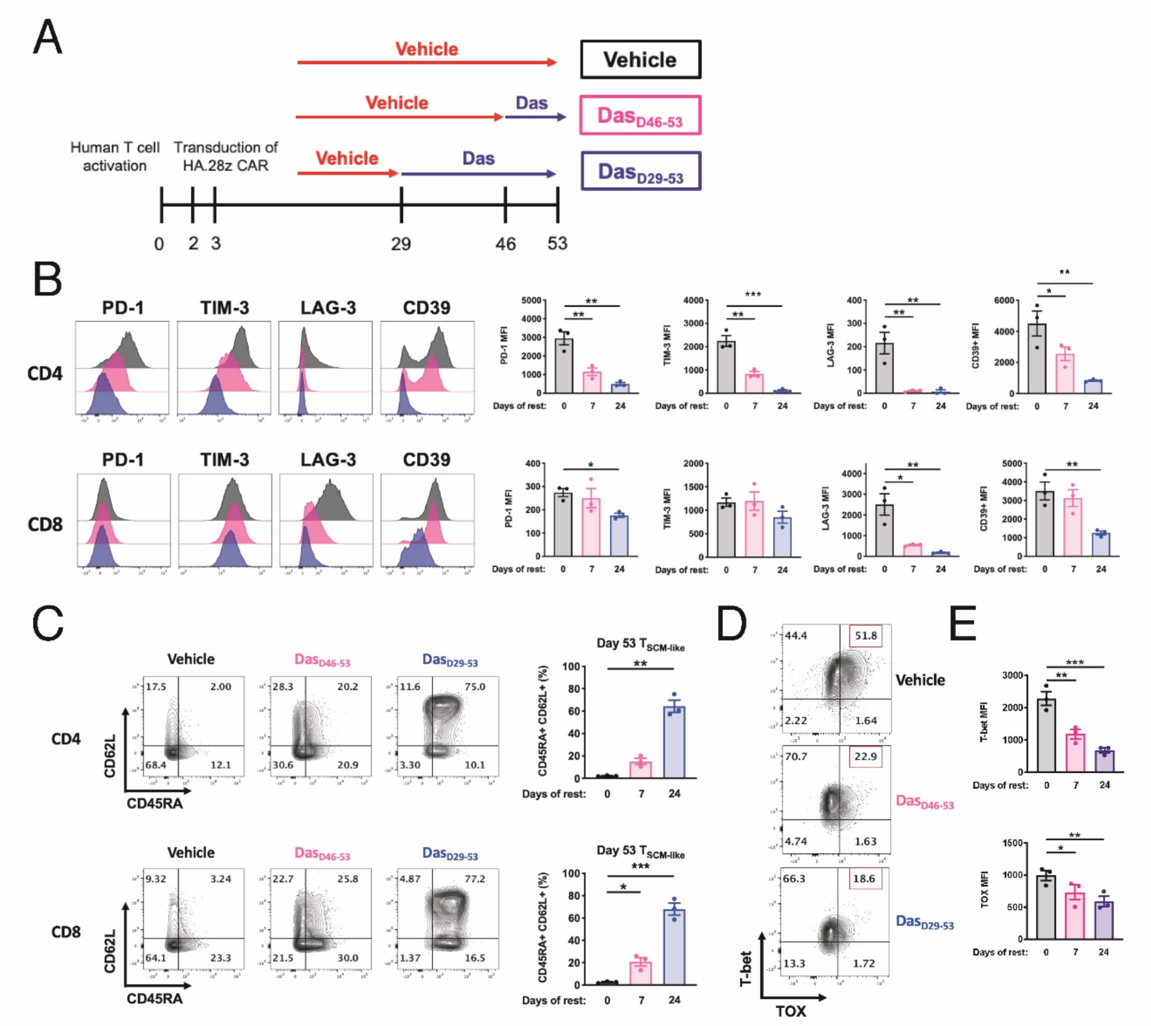
A
Vehicle
Vehicle
Das
Vehicle
Das
Human T cell activation
Transduction of HA.28z CAR
Vehicle
Das
Vehicle
DasD46-53
DasD29-53
0 2 3 29 46 53
B
PD-1
TIM-3
LAG-3
CD39
CD4
CD8
Days of rest:
C
Vehicle
DasD46-53
DasD29-53
CD62L
CD45RA
Day 53 TSCM-like
CD45RA+ CD62L+ (%)
D
Vehicle
T-bet
TOX
E
T-bet MFI
TOX MFI

Figure S10: Rested CAR-T cells resist the reacquisition of an exhaustion phenotype. **A-E)** HA.28ζ CAR-T cells were (A) cultured with vehicle (DMSO), dasatinib for 7 days (pink), or a pulse of dasatinib for 4 days followed by vehicle for 3 days (orange). Dasatinib was removed from the culture medium approximately 16 hours prior to in vitro tumor challenge on D18. **(B)** Change in percent CD8+ CAR-T cell expansion from D11 to D18 as measured by flow cytometry. **(C)** D18 exhaustion marker expression via flow cytometry. **(D)** D18 Incucyte assay (1:4 E:T). **(E)** IL-2 and IFNγ secretion on D18. Nalm6-GD2 (D and E) or 143B-GL (E) were used for co-culture assays. C shows mean ± SEM of n=3 individual donors. D shows mean ± SD of triplicate wells from 1 representative donor (n=3 individual donors). E shows D18 IL-2 and IFNγ secretion normalized to samples treated with dasatinib from D4-18, where error bars represent mean ± SEM of 3 individual donors. **F-J)** HA.28ζ.FKBP CAR-T cells were (F) continuously cultured with shield-1 (black), rested in the absence of shield-1 from D11-18 (pink), or rested in the absence of shield-1 for 4 days followed by addition of shield-1 for 3 days (orange). Shield-1 was added to all conditions 16 hours prior to in vitro tumor challenge on D18. **(G)** Change in percent CD8+ CAR-T cell expansion from D11 to D18 as measured by flow cytometry. **(H)** D18 exhaustion marker expression via flow cytometry. **(I)** D18 Incucyte assay (1:4 E:T). **(J)** IL-2 and IFNγ secretion on D18. Nalm6-GD2 (I) or 143B-GL (J) were used for co-culture assays. (H) shows mean ± SEM of 5 individual donors, (I) shows mean ± SD of triplicate wells from 1 representative donor (n=5 individual donors), and (J) shows mean ± SEM of 3 individual donors. Statistics were calculated using one- (C and H) or two-way (B, E, G, J, and D and I, last time point)

ANOVA and Dunnett's multiple comparisons test or Friedman test and Dunn's multiple comparisons test (C, LAG-3). *, $p<0.05$; **, $p<0.01$; ***, $p<0.001$; ****, $p<0.0001$

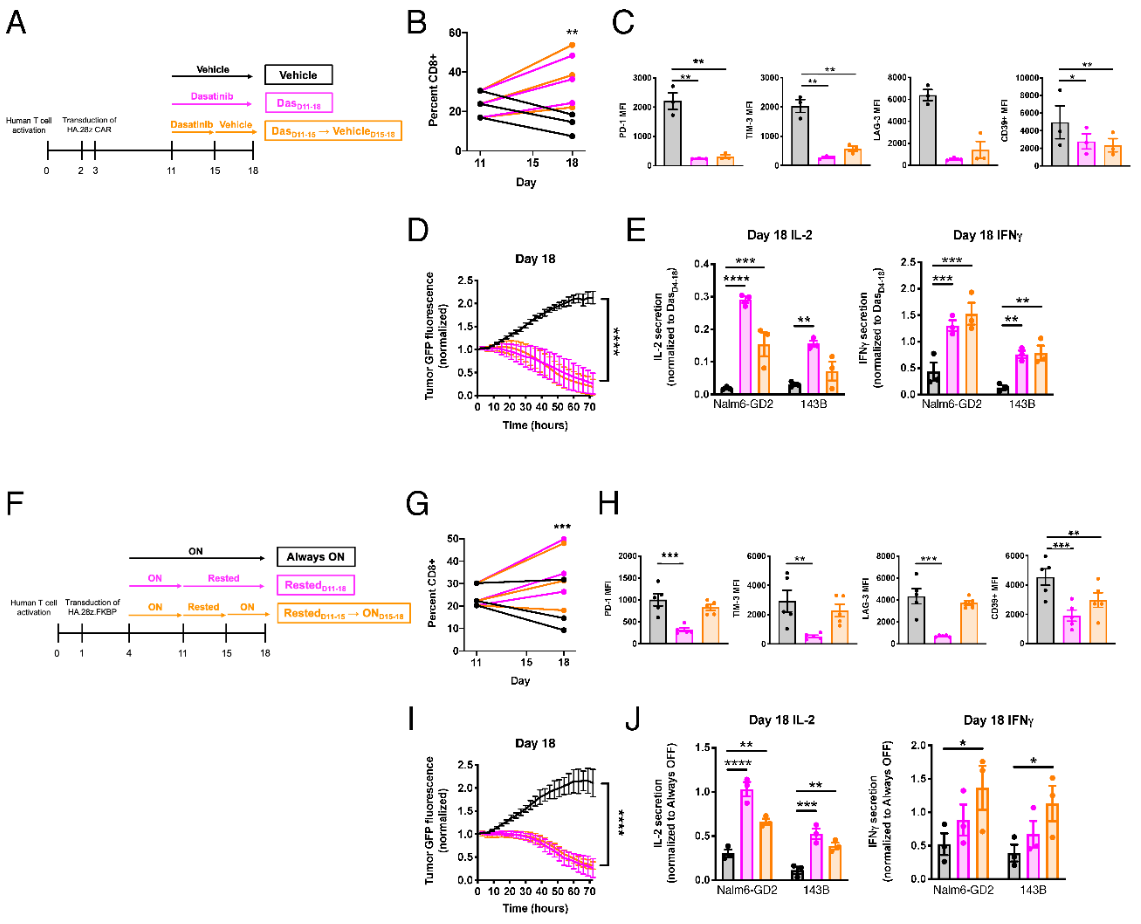
A
B
C
D
E
F
G
H
I
J
Human T cell activation
Transduction of HA.28ζ CAR
Vehicle
Dasatinib
Dasatinib Vehicle
Percent CD8+
Day
Day 18
Tumor GFP fluorescence (normalized)
Time (hours)
Day 18 IL-2
Day 18 IFNγ
Nalm6-GD2
143B
Transduction of HA.28z.FKBP
ON
Rested
Always ON
IL-2 secretion (normalized to Always OFF)
IFNγ secretion (normalized to Always OFF)

Figure S11: Rest reverses hallmarks of CAR-T cell exhaustion in vivo and enhances anti-tumor efficacy. A-B) Nalm6-bearing mice infused with CD19.BBζ CAR-T cells were treated with vehicle, dasatinib every other day, or dasatinib in a 3 day das/ 4 day OFF schedule for two weeks post-CAR-T cell infusion. (A) shows tumor bioluminescence at multiple time points from 1 representative experiment (n=2 independent experiments) and (B) shows survival data of n=9-10 mice/group from 2 independent experiments ($p<0.0001$ log-rank Mantel-Cox test). **C-D)** 143B-bearing mice infused with GD2.BBζ CAR-T cells were treated with vehicle or pulsed with dasatinib in a 3 day das/ 4 day OFF schedule. (C) shows tumor growth at multiple time points from 1 representative experiment (n=3 independent experiments) and (D) shows survival data of n=15 mice/group from 3 independent experiments ($p<0.0001$ log-rank Mantel-Cox test). **E-F)** $1x10^6$ 143B-GL osteosarcoma cells were engrafted in mice on day 0 and $10x10^6$ GD2.BBζ CAR-T cells expanded for 11 days in vitro were infused IV on day 3 post-engraftment. Mice were dosed with 50mg/kg dasatinib or vehicle twice per day from D12-18 or D16-18 post-engraftment, and tumor-infiltrating CAR-T cells (CAR-TIL) were isolated on D19. **E)** Tumor growth. Data shows mean ± SEM of 5 mice/group. **F)** CAR-TIL exhaustion marker and CD62L expression. Histograms show 1 representative mouse and MFI quantification shows mean ± SEM of n=5 mice per group. **G)** CD107a and TNFα gating strategy used to generate quantitative FACS data shown in Figure 7I (representative mouse from n=5 mice/group). Statistics were calculated using one- (F, PD-1, LAG-3, CD62L) or two-way ANOVA (E, Day 19) and Dunnett's multiple comparisons test or Kruskal-Wallis test and Dunn's multiple comparisons test (F, TIM-3 and CD39). *, $p<0.05$; **, $p<0.01$; ns, $p>0.05$

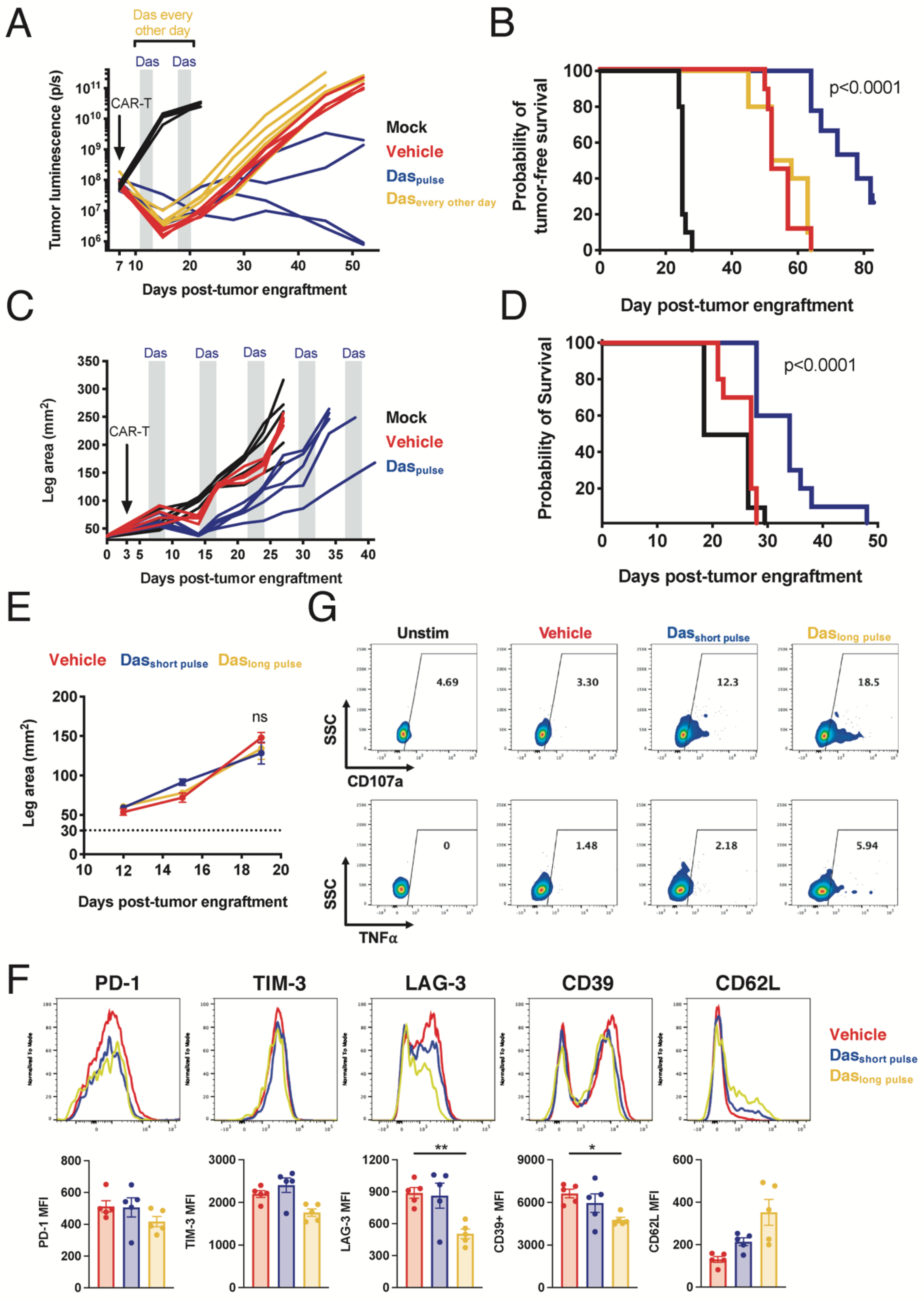
A
Das every other day
Das
Das
CAR-T
Tumor luminescence (p/s)
Days post-tumor engraftment
Mock
Vehicle
Das pulse
Das every other day
B
Probability of tumor-free survival
p<0.0001
Day post-tumor engraftment
C
Leg area (mm2)
CAR-T
Das
Days post-tumor engraftment
Mock
Vehicle
Das pulse
D
Probability of Survival
p<0.0001
Days post-tumor engraftment
E
Vehicle
Das short pulse
Das long pulse
Leg area (mm2)
ns
Days post-tumor engraftment
G
Unstim
Vehicle
Das short pulse
Das long pulse
SSC
CD107a
4.69
3.30
12.3
18.5
SSC
TNFα
0
1.48
2.18
5.94
F
PD-1
TIM-3
LAG-3
CD39
CD62L
Vehicle
Das short pulse
Das long pulse
PD-1 MFI
TIM-3 MFI
LAG-3 MFI
CD39+ MFI
CD62L MFI
**
*

Table S1: Antibodies and reagents.

REAGENT OR RESOURCE	SOURCE	IDENTIFIER
**Mass Cytometry Antibodies and Reagents**		
Anti-cPARP (clone Asp214)	BD	300443
Anti-CD27 (clone 323)	Biolegend	302839
Anti-CD62L (clone DREG-56)	Biolenged	304835
Anti-CD4 (clone RPT-T4)	Fluidigm	3145001B
Anti-CD8 (clone RPT-T8)	Fluidigm	3146001B
Anti-CD28 (clone CD28.2)	Biolegend	302937
Anti-PDL1 (clone 29E.2A3)	Fluidigm	3148017B
Anti-CD45RO (clone UCHL1)	Fluidigm	3149001B
Anti-OX-40 (clone ACT35)	Fluidigm	3150023B
Anti-2B4 (clone C1.7)	Biolegend	329502
Anti-TIM-3 (clone F38-2E2)	Fluidigm	3153008B
Anti-CXCR3 (clone G025H7)	Fluidigm	3156004B
Anti-4-1BB (clone 4B4-1)	Fluidigm	3158013B
Anti-Blimp-1 (clone UCHT1)	Biolegend	300402
Anti-T-bet (clone 4B10)	Biolegend	3160010B
Anti-CTLA-4 (clone 14D3)	Fluidigm	3161004B
Anti-CD25 (clone M-A251)	BD	555430
Anti-BTLA (clone MIH26)	Fluidigm	3163009B
Anti-CD39 (clone A1)	Biolegend	328221
Anti-LAG-3 (clone 874501)	Fluidigm	3165028B
Anti-CCR7 (clone G043H7)	Fluidigm	3167009A
Anti-IL-7R (clone A019D5)	Fluidigm	3168017B
Anti-CD45RA (clone HI100)	Fluidigm	3170010B
Anti-Ki-67	Biolegend	350523
Anti-CD95 (clone DX2)	Biolegend	305631

Anti-PD-1 (clone EH12.2H7)	Fluidigm	3174020B
Anti-Eomes (clone WD1928)	eBioscience	14-4877-82
DNA intercalator	Fluidigm	201191B
Cisplatin	Fluidigm	201064
Cell-ID 20-Plex Pd Barcoding Kit	Fluidigm	201060
EQ Four Element Calibration Beads	Fluidigm	201078
**Flow Cytometry Antibodies and Reagents**		
Anti-14g2A idiotype antibody (clone 1A7)	NIH	
Anti-CD4 (clone SK3)	BD	563550
Anti-CD8 (clone SK1)	BD	564912
Anti-PD-1 (clone eBioJ105)	eBioscience	25-2799-42
Anti-TIM-3 (clone F38-2E2)	Biolegend	345030
Anti-LAG-3 (clone 3DS223H)	eBioscience	12-2239-42
Anti-CD39 (clone A1)	Biolegend	328226
Anti-CD62L (clone DREG-56)	BD	562719
Anti-CCR7 (clone 150503)	BD	562555
Anti-CD45RO (clone UCHL1)	BD	25-0457-42
Anti-CD45RA (clone L48)	BD	649458
Anti-CD127 (clone A019D5)	Biolegend	351310
Anti-T-bet (clone 4B10)	Biolegend	644819
Anti-LEF1 (clone C12A5)	Cell Signaling	14440S
Anti-TCF7 (clone C63D9)	Cell Signaling	6709S
Anti-TOX (clone REA473)	Miltenyi Biotec	130-118-335
Anti-IL-2 (clone MQ1-17H12)	Biolegend	500326
Anti-IFNy (clone 4S.B3)	Biolegend	502530
Anti-TNFa (clone MAb11)	Biolegend	502940
Anti-CD69 (FN50)	Biolegend	310930

Anti-CD107a (clone H4A3)	Biolegend	328634
Monensin	eBioscience	00-4505-51
Fixable Viability Dye	eBioscience	65-0866-14
FoxP3 Transcription Factor Staining Buffer Set	eBioscience	00-5523-00
CountBright Absolute Counting Beads	Thermo Scientific	C36950
DyLight 650 Microscale Antibody Labeling Kit	Thermo Fisher	84536
DyLight 488 Microscale Antibody Labeling Kit	Thermo Fisher	53025
